# Development and Large-Scale Testing of a Novel One-Step Triplex RT-qPCR Assay for Simultaneous Detection of “Neurotropic” Porcine Sapeloviruses, Teschoviruses (*Picornaviridae*) and Type 3 Porcine Astroviruses (*Astroviridae*) in Various Samples including Nasal Swabs

**DOI:** 10.3390/v14030513

**Published:** 2022-03-02

**Authors:** Zoltán László, Péter Pankovics, Gábor Reuter, Attila Cságola, Kornélia Bodó, Gábor Gáspár, Mihály Albert, Hunor Bíró, Ákos Boros

**Affiliations:** 1 Department of Medical Microbiology and Immunology, Medical School, University of Pécs, 7624 Pécs, Hungary; laszlo.zoltan2@pte.hu (Z.L.); pankovics.peter@pte.hu (P.P.); reuter.gabor@pte.hu (G.R.); gaspgabor@gmail.com (G.G.); 2 Ceva Phylaxia Ltd., 1107 Budapest, Hungary; attila.csagola@ceva.com (A.C.); mihaly.albert@ceva.com (M.A.); 3 Department of Immunology and Biotechnology, Clinical Center, Medical School, University of Pécs, Szigeti u, 12, 7643 Pécs, Hungary; kbodo@foss.dk; 4 SHP Ltd., 7400 Kaposvár, Hungary; hunor.biro@t-online.hu

**Keywords:** picornavirus, sapelovirus, teschovirus, astrovirus, meningoencephalomyelitis, swine, RT-qPCR, nasal, epidemiology

## Abstract

Porcine sapeloviruses, teschoviruses of family *Picornaviridae* and type 3 porcine astroviruses of family *Astroviridae* are (re-)emerging enteric pathogens that could be associated with severe, disseminated infections in swine, affecting multiple organs including the central nervous system (CNS). Furthermore, small-scale pioneer studies indicate the presence of these viruses in porcine nasal samples to various extents. The laboratory diagnostics are predominantly based on the detection of the viral RNA from faecal and tissue samples using different nucleic-acid-based techniques such as RT-qPCR. In this study, a novel highly sensitive one-step triplex RT-qPCR assay was introduced which can detect all known types of neurotropic sapelo-, tescho- and type 3 astroviruses in multiple types of samples of swine. The assay was evaluated using in vitro synthesized RNA standards and a total of 142 archived RNA samples including known sapelo-, tescho- and type 3 astrovirus positive and negative CNS, enteric and nasal specimens. The results of a large-scale epidemiological investigation of these viruses on *n* = 473 nasal swab samples from *n* = 28 industrial-type swine farms in Hungary indicate that all three neurotropic viruses, especially type 3 astroviruses, are widespread and endemically present on most of the investigated farms.

## 1. Introduction

Porcine sapeloviruses (PSV/SV-A) and porcine teschoviruses (PTV/TV-A) of family *Picornaviridae* and type 3 porcine astroviruses (PoAstV-3) of family *Astroviridae* are increasingly recognized enteric pathogens associated with a variety of diseases including severe central nervous system disorders (CNS), with a high mortality rate in swine [1,2,3,4,5,6,7,8].

The currently known strains, up to 100 PSV, originated from more than 12 countries worldwide and are classified into two types (an officially recognized PSV-1 and a candidate type PSV-2) in the genus *Sapelovirus*, species *Sapelovirus A* [9,10,11]. In contrast, there are more than 17 types of PTVs and nearly 200 known strains in species *Teschovirus A* and *B*, genus *Teschovirus* [9,11]. Porcine astrovirus type 3 (tentatively called Mammastrovirus 22) is one of the five currently known, phylogenetically highly diverse porcine astrovirus types (PoAstV1-5). PoAstV-3 belongs to the human-mink-ovine (HMO) phylogenetic clade of genus *Mammastrovirus* along with other neurotrophic astroviruses [12].

Although all three viruses are able to cause asymptomatic infections, multiple types/strains of PTV (e.g., PTV-2, -3, -11, -13) [4,6,7] and PSV [1,3] are detected in various organs/samples (including CNS) from pigs showing CNS-related symptoms (paraplegia, paralysis, paresis and ataxia) with a high mortality rate. Interestingly, from the five known porcine astrovirus types, only type 3 was conclusively detected from CNS samples of swine with signs of meningoencephalomyelitis and, therefore, could have neurotropic potential [12]. Besides the CNS and other internal organs, these three viruses have already been detected in respiratory/nasal samples as well with relatively low copy numbers [5,10,13,14,15,16], but large-scale epidemiological studies of these viruses in nasal swabs have not been performed previously.

All three viruses have single-stranded RNA genomes with positive polarity. The genomes are started with highly structured 5′ untranslated regions (UTRs) and are terminated with a 3′UTR and a poly(A)-tail. PTVs and PSVs have a single open reading frame (ORF) which encodes a leader peptide (L) followed by capsid proteins (VP4-VP2-VP3-VP1) and the non-structural (2A-2B-2CHel-3A-3B-3CPro-3DRdRp, Hel: helicase, Pro: protease, RdRp: RNA-dependent RNA polymerase) peptides [17,18]. The PoAstV-3 genome has three overlapping ORFs (ORF1a, ORF1b and ORF2); the ORF1a and the adjacent ORF1b could be transcribed continuously (ORF1ab), resulting in the production of nonstructural polyprotein 1ab (NSP-1ab), while ORF2 could be separately transcribed into capsid proteins [12,19]. RNA viruses, including astroviruses, can create novel, heterogenic strains employing recombination (RNA crossover), which preferentially takes place in certain “hot spots” of the genome, such as the junction of ORF1b/ORF2 [20]. There are some conserved genome parts in all three viruses, such as 5′UTR, 2C-Hel and 3D-RdRp in PSV&PTV or Hel-, Pro-, and RdRp-encoding regions of NSP-1ab, immediately followed by the conserved 5′-part of ORF2 (encoding the N-terminal assembly domain of the capsid) which represent suitable oligonucleotide primer target sites for PCR detections [10,21,22].

There are several PCR-based assays available for the specific singleplex (e.g., PTV: [23,24]; PSV: [25]; PoAstV-3: [26]) or duplex (e.g., PSV&PTV: [27]) detection of these viruses, but an assay for the simultaneous detection of all three viruses is currently unknown.

The purpose of this study was to develop an easy-to-use, fluorophore-tagged hydrolysis-probe-based one-step triplex RT-qPCR assay capable of the simultaneous detection of all known types of *Sapelovirus A*, *Teschovirus A/B* and the “neurotropic” PoAstV-3 in any types of samples of swine. The created singleplex and triplex assays were evaluated using in vitro synthesized RNA templates and *n* = 142 archived RNA samples (including CNS, other tissue, enteric and nasal samples) and its specificity was confirmed by direct Sanger-sequencing of selected RT-qPCR products. Epidemiological data originated from the large-scale RT-qPCR screening of *n* = 473 nasal swab samples collected from 21–25-day-old asymptomatic swine from *n* = 28 different, highly prolific, industrial-type swine farms are also summarized.

## 2. Materials and Methods

### 2.1. Sample Background and RNA Extraction

A total of 142 selected, archived total RNA samples (*n* = 100 faecal/rectal swabs, *n* = 1 urine and *n* = 41 tissue specimens) collected from asymptomatic (*n* = 92) or paraplegic (*n* = 20) swine including (but not limited to) previously known PSV, PTV and PoAstV-3 positive and negative samples were used for the comparison of diagnostic sensitivities of triplex RT-qPCR and formerly used RT-PCR screening assays (Table 1, Tables S1 and S2). Note that all archived RNA samples (including the previously tested specimens) were re-tested with PSV, PTV and PoAstV-3 screening RT-PCR assays prior to RT-qPCR reactions. Details of TRI Reagent-based total RNA extraction of these samples were described in our previous studies [5,10,28].

**Figure 1:**
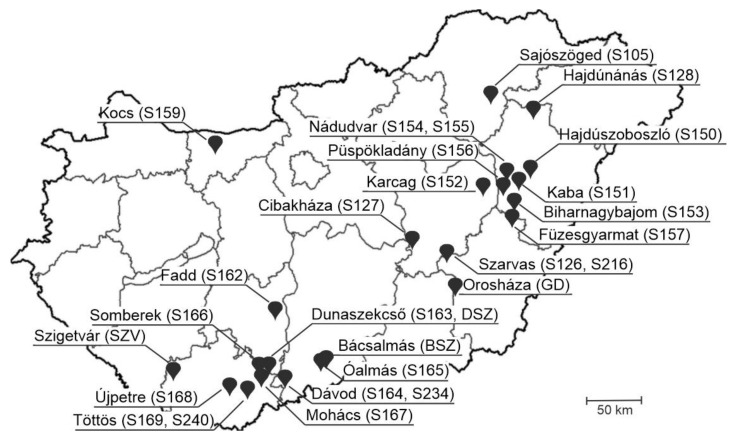
Localizations of *n* = 28 swine farms (with farm IDs) included in the investigation of the prevalence of porcine sapelovirus (PSV), porcine teschovirus (PTV) and porcine astrovirus type 3 (PoAstV-3) in *n* = 473 nasal swabs of 21–25-day-old asymptomatic swine by RT-qPCR.

In addition to the archived RNA samples, a total of 473 nasal swabs—which were collected at 28 different, highly prolific, industrial-type swine farms located in various geographical regions of Hungary (Figure 1)—were also screened by RT-qPCR (Table S3). The number of collected nasal swab samples/farms ranged between *n* = 9 and *n* = 42, but *n* > 10 samples were collected from the majority (*n* = 26/28, >92%) of the investigated farms (Table S3). Swabs were collected from 21–25-day-old asymptomatic swine using two, sterile polyester-tipped swabs and re-suspended in 500 µL sterile PBS.

Furthermore, a total of 21 tissue samples (brainstem *n* = 8, spinal cord *n* = 6, cerebellum *n* = 7) of paraplegic pigs (*n* = 8) with unknown etiology were also tested by our novel RT-qPCR assay (Table S4).

The tissue samples (average mass was 50–100 mg) were mechanically homogenized using manual Potter-Elvehjem tissue grinders (Sigma-Aldrich, St. Louis, MI, USA) prior to RNA isolation. Total RNA was extracted from 150 μL homogenates or re-suspended swab samples using TRI Reagent (MRC, Cincinnati, OH, USA) according to the manufacturer’s instructions. At the final step, the precipitated RNA was resolved with 100 µL of nuclease-free dH_2_O (NFW).

### 2.2. RT-PCR and Dye-Terminator Sequencing

The applied reaction conditions, screening/generic primer sets (Table S1) and reagents used for PSV, PTV or PoAstV-3-specific RT-PCR screening assays (i.e., conventional RT-PCR-based detection of a given virus with the use of virus-specific oligonucleotide primer pairs) were the same as described previously with only minor modifications [5,10,21,28]. Briefly, 5 μL total RNA sample was used in the 25 μL final volume of reverse transcription reactions. The total volume of the RT reaction was then used for PCR in a final volume of 50 μL. Selected PCR products/plasmid-inserts were sequenced directly using BigDye™ Terminator v1.1 Cycle Sequencing Kit (Thermo-Fisher, Waltham, MA, USA) according to the manufacturer’s instructions and run on an automated ABI 3500 Genetic Analyzer (Applied Biosystems/Hitachi, Tokyo, Japan). For the analyses of sequence data, Chromas Ver. 2.6.6, Geneious Prime ver. 2021.2.2 (Biomatters, Auckland, New Zealand) and National Centre for Biotechnology Information (NCBI) BLASTn web tool (https://blast.ncbi.nlm.nih.gov/Blast.cgi, accessed on 12 February 2021) were used.

### 2.3. Design of Oligonucleotide Primers and Hydrolysis Probes for the RT-qPCR-Based Detection of PTV, PSV and PoAstV-3

**Figure 2:**
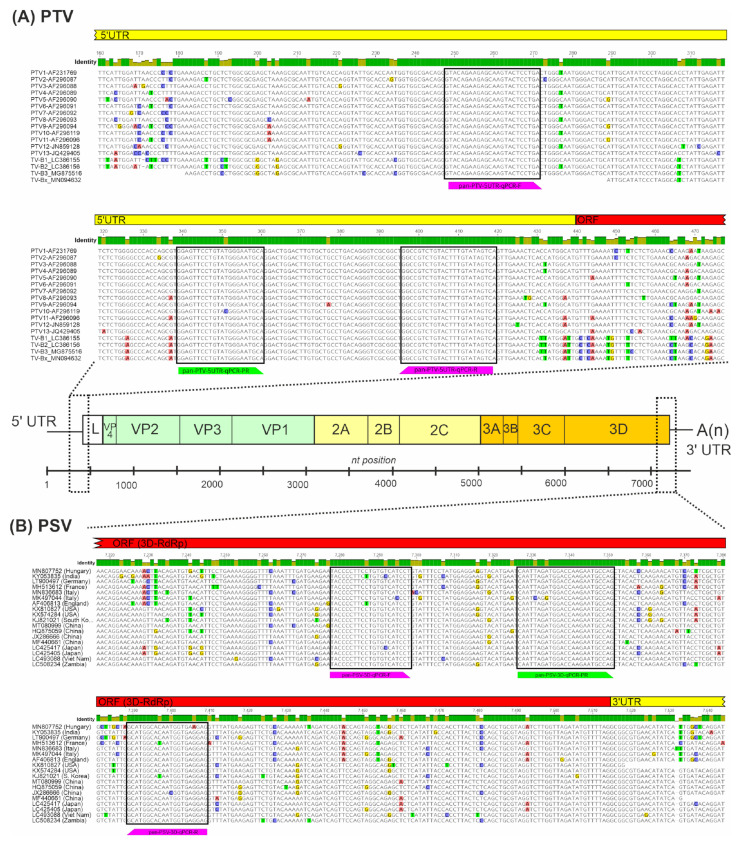
Partial 5′UTR-L and 3D^RdRp^-3′UTR nucleotide sequence alignments of representative members of *Teschovirus A* and *B* (**A**) and *Sapelovirus A* (**B**) with the binding sites of oligonucleotide primers (magenta) and probes (green) used in singleplex/triplex RT-qPCR assays of this study. The locations of the aligned regions and primer–probe binding sites are marked with dotted lines in a schematic picornavirus genome map. The identity graphs above the alignments show the identical (green bars) and moderately variable (pale yellow bars) genomic regions. The countries of origins of different *Sapelovirus A* sequences are found in brackets after the accession numbers.

All complete nucleotide (nt) genomic sequences of PTVs, PSVs and PoAstV-3s were downloaded from the National Centre for Biotechnology Information (NCBI) nucleotide database (accessed on 25 January 2021) then aligned separately using the MUSCLE alignment tool of Geneious Prime ver. 2021.2.2 (Biomatters, Auckland, New Zealand) [29]. After the identification of conserved genome regions (5′UTR for PTV, 3D-RdRp for PSV and ORF1ab-ORF2 junction for PoAstV-3) suitable for virus-specific primer–probe design, the alignments were then supplemented with additional available partial 5′UTR (PTV), 3D-RdRp (PSV) and ORF1ab-ORF2 (PoAstV-3) sequences, and the resulting expanded alignments were used for the final design of oligonucleotide primers and probes with melting temperatures (Tm) ranging between 64 and 65 °C (Figure 1 and Figure 2; Table 1). The designed primer and probe sequences were compared to the non-redundant database of GenBank by BLASTn-search to in silico rule out non-specific binding.

For multiplexing purposes, the 5′ fluorophores of three hydrolysis probes with non-overlapping peak emission wavelengths which are compatible with the CFX96 Touch Real-Time PCR Detection System (Bio-Rad Laboratories, Inc., Hercules, CA, USA) and adequate quenchers were selected using PrimeTime^®^ Multiplex Dye Selection Tool of Integrated DNA Technologies (IDT, USA, https://eu.idtdna.com/site/order/qpcr/primetimeprobes/multiplex, accessed on 12 February 2021) (Table 1). The designed primers and probes (either separately or as an assay-specific mixture) were synthesized and shipped lyophilized by IDT.

Oligonucleotides were re-suspended with NFW to generate stocks of either 50 µM (separate primers) and 25 µM (separate probes) or 40× (assay-specific mixtures) which were divided into smaller (30–50 µL) aliquots and stored in the dark at −20 °C until use. The 40× diluted assay-specific mixtures contained 20 µM primers and 10 µM probes.

### 2.4. Production of PSV, PTV and PoAstV-3 RNA Standards

The target virus-specific RNA standards were prepared in vitro from short (406–591 bp long) PCR-products that contained the binding sites of primers and probes to optimize the RT-qPCR reaction conditions and the assay performance. For details of the production of virus-specific RNA standards, see the technical appendix [30]. Briefly, the generated PCR products were cloned into DH5-Alpha-type competent *Escherichia coli* cells (Thermo-Fisher) using T7-promoter sequence-containing pCR2.1 vectors of TA Cloning^®^ Kit (Thermo-Fisher). The generated purified plasmids were digested with the appropriate restriction enzymes and the resulting linearized products were used as templates for in vitro RNA synthesis using TranscriptAid T7 High Yield Transcription Kit (Thermo-Fisher). The T7 reaction products were digested with TURBO™ DNase enzyme and purified by TRI^®^ Reagent (MRC, Cincinnati, OH, USA). The copy number of the produced viral RNA standards were calculated and ten-fold serial dilutions of mixed viral RNA standards (from 2 × 10^9^ to 2.0 copies/µL of each element of the mixture) were created using NFW.

### 2.5. RT-qPCR and Data Analyses

For all RT-qPCR reactions, LightCycler^®^ Multiplex RNA Virus Master One-step RT-qPCR Master Mix (Roche, Switzerland) was used according to the manufacturer’s instructions with minor modifications. Based on preliminary test results, the amount of reverse transcriptase was increased from 0.1 µL/reaction to 0.15 µL/reaction, and 40× assay-specific mixtures (primer–probe mixtures, 0.5 µL/reaction) were used instead of 20× stocks, resulting in the final concentrations of 500 nM of all primers and 250 nM of each of the probes in a final volume of 20 µL. The amount of RNA samples was always 5 µL.

All the reactions were conducted on either 8-well white strips (Bio-Rad Laboratories, Inc., Hercules, CA, USA) or 96-well white plates (BIOplastics, Landgraaf, The Netherlands) and run on a CFX96 Touch Real-Time PCR Detection System (Bio-Rad Laboratories, Inc., Hercules, CA, USA). The thermal program consisted of the following steps: reverse transcription at 50 °C for 10 min, pre-incubation at 94 °C for 3 min, amplification (at 94 °C for 15 s and at 60 °C for 30 s) × 44 repeats, and final cooling at 40 °C for 30 s. The detection of the fluorescence signals in three channels (FAM, VIC and Cy5) took place at the end of each amplification step. NFW was used as a non-template control. The data analyses were conducted with the Bio-Rad CFX Maestro 2.2 ver. 5.2.008.0222 software (Bio-Rad Laboratories, Inc., Hercules, CA, USA), applying automatic baseline detection and manual thresholds of 430, 151 and 110 for PSV, PTV and PoAstV-3, respectively. The amplification efficiencies, slope and correlation coefficient values were calculated automatically by the software.

### 2.6. Analytical Performance Assays and Statistical Calculations

Analytical sensitivity and specificity tests of singleplex/triplex RT-qPCR assays were carried out using 10-fold serial dilutions of mixed PSV/PTV/PoAstV-3 RNA standards, ranging from final concentrations of 1 × 10^8^ to 1 × 10^1^ copies/reaction/standards.

Lower limit of detection (LOD) analyses of singleplex and triplex RT-qPCR assays were conducted using four final concentrations of mixed PSV/PTV/PoAstV-3 RNA standards (1 × 10^4^, 1 × 10^3^, 1 × 10^2^ and 1 × 10^1^ copies/reaction). The test, which consisted of all three singleplex and a triplex assay in three technical replicates in a 96-well plate format, was repeated four (1 × 10^1^, 1 × 10^2^, 1 × 10^3^) or two (1 × 10^4^) times.

Intra-assay reproducibility (coefficients of variation, CV%) was tested using two final concentrations of the mixed PSV/PTV/PoAstV-3 RNA standards (1 × 10^3^ and 1 × 10^2^ copies/reaction/standard) in three technical replicates both in singleplex and triplex formats. Inter-assay CV% were calculated based on the results of four independent RT-qPCR runs containing all three singleplex and triplex assays in a 96-well plate format on different days by two different people. Only Cq values from samples with normal, sigmoidal-shaped amplification curves were used in the calculations. The CV% values were calculated by dividing the standard deviation quantities by the corresponding mean Cq-s of the groups of replicates (i.e., 1 × 10^2^ or 1 × 10^3^ in singleplex or triplex assays) and represented as percentages. The diagrams and statistical calculations (standard deviation, mean, coefficient of variation%) were conducted in Excel of Microsoft Office Professional Plus 2016. For data visualization, CorelDraw standard 2020 software ver. 22.0.0.474 was used. Box plot/bean-plot diagrams of measured Cq values were created using BoxPlotR web tool [31]. The reagents, qPCR plastics, reaction conditions and data analyses were identical in all experiments, as described in the RT-qPCR and data analyses section.

## 3. Results

### 3.1. Design of Oligonucleotide Primers and Hydrolysis Probes for PTV, PSV and PoAstV-3

**Figure 3:**
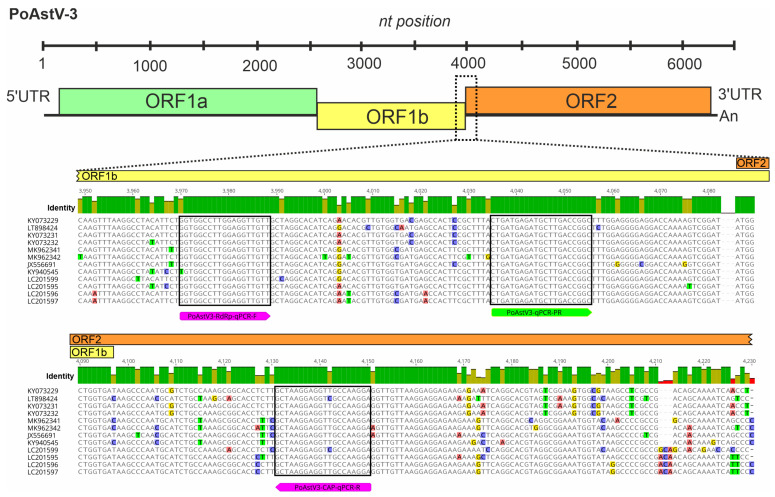
Nucleotide sequence alignment of the partial ORF1b-ORF2 junction of all publicly available porcine astrovirus type 3 (PoAstV-3) viruses with the binding sites of oligonucleotide primers (magenta) and a probe (green) used in singleplex/triplex RT-qPCR assay of this study. The location of the aligned region and primer–probe binding sites are marked with dotted lines in the schematic astrovirus genome map. The identity graph above the alignment shows the identical (green bars) highly (red bars) and moderately variable (pale yellow bars) genomic regions.

Based on the generated nt alignments of known PTV, PSV and PoAstV-3 sequences, virus-specific primers and three different 5′ fluorophore-tagged (SUN for PTV and 6-FAM for PSV Cy5 for PoAstV-3) hydrolysis probes were designed manually to selected, highly conserved genomic regions, such as the 5′UTR (PTV), 3D-RdRp (PSV) (Figure 2, Table 1). To ensure the specific detection of PoAstV-3 and no other PoAstV types (including potential ORF1b/ORF2 recombinants), the PoAstV-3 primer–probe set was specifically designed to the junction of ORF1b (RdRp)/ORF2 (Figure 3, Table 1). The designed primer–probe sets are theoretically able to detect all the known PTV, PSV and PoAstV-3 viruses, regardless of the geographical origins, with only 1–2 mismatches between primer–probe and target sequences (Figure 2 and Figure 3).

Furthermore, the primer–probe sets were specifically designed to generate PCR products suitable for direct sequencing (between the lengths of 100 and 200 bp, Table 1) without compromising the quantification efficiency by the use of larger amplicons [32].

### 3.2. Performance and Analytical Sensitivity of Singleplex and Triplex RT-qPCR Assays

Based on the distribution of Cq values (ranging between 33.30 and 37.03 in PSV, 32.79 and 36.71 in PTV, 33.63 and 38.37 in PoAstV-3) measured at the lowest analyzed concentration of mixed PTV, PSV and PoAstV-3 RNA standards (1 × 10^1^ copies/reaction), the cut-off Cq (36.5) was arbitrarily set to be located uniformly between the highest and second highest Cq values in the case of all three viruses (Figure S1). This cut-off value was applied in all the further reactions.

**Figure 4:**
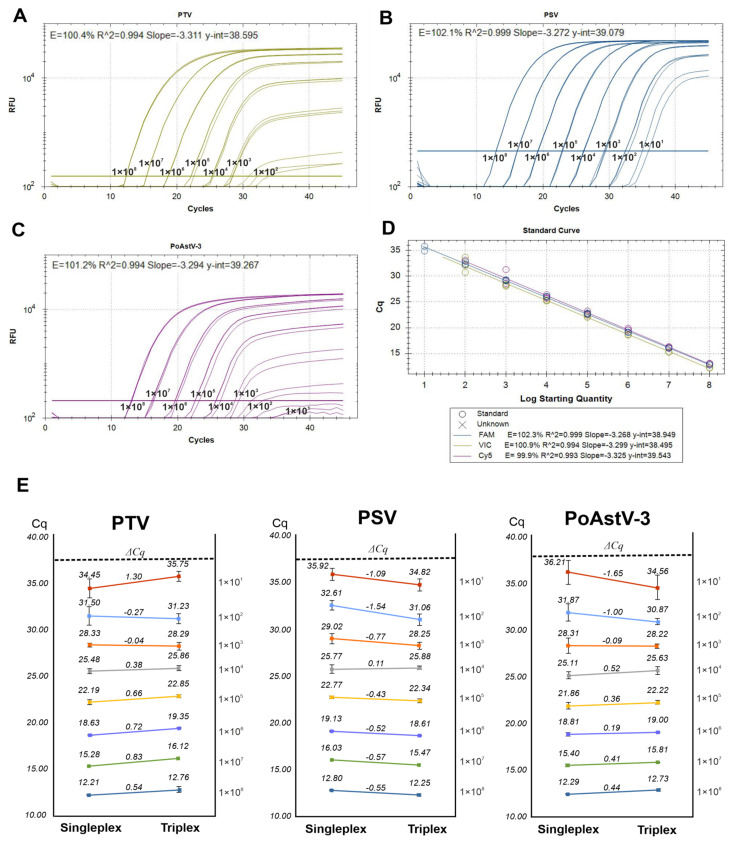
Amplification plots and standard curves of singleplex PTV (**A**), PSV (**B**) and PoAstV-3 (**C**) and triplex (**D**) assays using 10-fold serial dilutions of mixed PSV, PTV and PoAstV-3 RNA standards as templates (1 × 10^8^ to 1 × 10^1^ copies/reaction). Each dilution had triple technical replicates. The amplification efficiencies (**E**), slope, correlation coefficient (R^2^) and Y-intercept (y-int) values were calculated automatically by the Bio-Rad CFX Maestro 2.2 ver. 5.2.008.0222 software. RFU: relative fluorescence units. (**E**) Summary of measured Cq values and Cq differences (ΔCq) based on the comparisons of singleplex and triplex assays using the same 10-fold serial dilutions of mixed PSV, PTV and PoAstV-3 RNA standards as templates (1 × 10^8^ to 1 × 10^1^ copies/reaction).

The analytical sensitivity and specificity of the RT-qPCR assays were first tested separately in singleplex reactions using serial dilutions (1 × 10^8^ – 1 × 10^1^ copies/reaction) of mixed PTV, PSV and PoAstV-3 RNA standards (Figure 4A–C), then in triplex format (Figure 4D) using the same reaction conditions. To rule out any false-positive reactions, separate PTV, PSV and PoAstV-3 RNA standards were tested in independent RT-qPCR reactions with all primer–probe combinations. Signals were detectable only in those of the reactions when a given standard RNA and its specific primer–probe counterparts were used. The slope, correlation coefficient (R^2^) and reaction efficiency (E) values of each singleplex and triplex run are found in Figure 4A–D.

In both the singleplex and triplex assays, the lowest tested concentration showing signal above the threshold was 1 × 10^1^ (10 copies/reaction); although, in this concentration, the measured Cq values showed relatively high variations and only 36.0–82.0% of the reactions gave detectable signals (Figure 4 and Figure S1, Table 2). Nevertheless, 1 × 10^2^ (100 copies/reaction) concentration was detectable in more than 95% of the repeats of all three targets (Table 2), which suggest that the lower limit of detection of the singleplex/triplex assays are around 100 copies.

The coefficients of variation (CV%) values were calculated from the results of four independent singleplex and triplex assays, which were run on different days by two people applying two selected dilutions of mixed RNA standards (1 × 10^2^ and 1 × 10^3^ copies/reaction) in each run. The CV% values of intra- and inter-assays ranged between 0.03–4.62% and 0.86–3.29%, respectively, which indicate the relatively high repeatability for both singleplex and triplex PCR assays (Table 3).

### 3.3. Comparison of Triplex RT-qPCR and RT-PCR Assay Diagnostic Sensitivity in Selected Samples

To analyze the clinical performance of the triplex RT-qPCR assay and to compare it with previously used RT-PCR screening assays, a total of 142 archived RNA samples of swine (faecal/rectal swabs *n* = 100, urine *n* = 1 and tissue specimens *n* = 41) were selected, including (but not limited to) previously known PSV, PTV and PoAstV-3 positive and negative samples (Table S2). The generic primer sets used for the RT-PCR screening reactions are targeting similar genomic regions (5′UTR of PTVs, 3D-RdRp of PSVs and ORF1ab of PoAstV-3s) as the primers of the assays (Table 1 and Table S1) [5,10,21,28].

The triplex RT-qPCR assay identified 64 (57 + 7) PSV, 47 (42 + 5) PTV and 20 (18 + 2) PoAstV-3 positive samples with the Cq values ranged between 18.55–36.37 of PSV, 18.39–32.10 of PTV and 14.60–29.44 of PoAstV-3, respectively (Table 4 and Table S2). Double infections were identified in 26.0% (PSV&PTV *n* = 26/100) and 1.0% (PSV&PoAstV-3 *n* = 1) of enteric, while 9.8% (PSV&PTV *n* = 4/41, 2 intestinal and 2 mesenteric lymph node) and 4.9% (PTV&PoAstV-3 *n* = 2/41, a nasal mucosa and a tonsil sample) of tissue samples (Table S2). Triple infections were not identified.

A total of 63 (57 + 6) PSV, 49 (42 + 7) PTV and 25 (18 + 7) PoAstV-3 positive samples were identified by RT-PCR screening reactions (Table 4 and Table S2). The agreements of the two assays were calculated as 90.85%, 91.55%, and 93.66% in the mutual detection of PSV, PTV and PoAstV-3-s, respectively (Table 4). The PCR product of ambiguous samples (either RT-qPCR +/RT-PCR − or RT-qPCR −/RT-PCR +) from both types of assays were all sequenced directly by Sanger-sequencing to rule out false-positive samples (data not shown). The results of the sequencing indicated the presence of 2/3, 1/3 and 0/5 false positive (either non-viral sequences or non-sequenceable) samples of PSV, PTV and PoAstV-3 identified by triplex RT-qPCR/RT-PCR assays, respectively (Table 4 and Table S1). The clinical sensitivity and specificity percentages of triplex RT-qPCR assay are found in Table 4. The samples used for sensitivity and specificity calculations have either identical results in both assays (+/+ or −/−, i.e., true positives and true negatives) or confirmed/disproved by sequencing (i.e., false negatives/positives) (Table 4 and Table S1).

### 3.4. Investigation of Nasal Swab and Central Nervous System Samples by RT-qPCR

**Figure 5:**
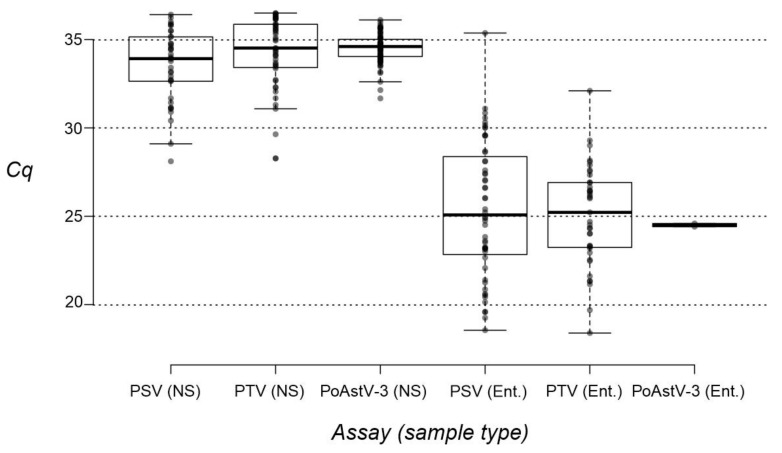
Box plot of measured Cq values of nasal swabs (NS) and faecal/rectal swab samples (Ent.) of porcine sapelovirus (PSV), porcine teschovirus (PTV) and porcine astrovirus type 3 (PoAstV-3). Center lines show the medians; box limits indicate the 25th and 75th percentiles as determined by R software; whiskers extend 1.5 times the interquartile range from the 25th and 75th percentiles; outliers are represented by dots; data points are plotted as gray circles. *n* = 37, 47, 84, 52, 39, and 2 sample points.

The presence of PSV, PTV and PoAstV-3 was investigated by our RT-qPCR assays in *n* = 473 nasal swab samples collected from 21–25–day–old asymptomatic swine from *n* = 28 different Hungarian swine farms (Figure 1, Table 5 and Table S3). While PSV and PTV could be identified in 7.8% (37/473 positive) and 9.9% (47/473 positive) of the investigated samples, PoAstV-3 positivity rate was measured to be much higher (17.8%, 84/473 positive) (Table S3). Double infections were identified in 1.3% (PSV&PoAstV-3, *n* = 6), 1.5% (PSV&PTV, *n* = 7) and 2.8% (PTV&PoAstV-3, *n* = 13) of the nasal samples, while triple infections were identified in only 0.4% (*n* = 2) of the cases (Table 5). The Cq values ranged between 28.11–36.41, 28.27–36.50 and 31.67–36.11 of PSV, PTV and PoAstV-3 in nasal swabs, respectively (Table S3). For comparison, the lowest Cq values measured in enteric samples were 18.55 (PSV), 18.39 (PTV) and 24.41 (PoAstV-3) (Table S2). Additionally, the comparison of Cq value distributions measured in nasal swabs and faecal/rectal swabs of all three viruses also indicate the overall lower Cq values measured in enteric than nasal samples (Figure 5).

At the farm level, while there are 67.9% (*n* = 19) and 42.9% (*n* = 12) of the 28 investigated farms where no PSV and PTV were detectable in nasal swabs until then there were only *n* = 4 PoAstV-3 negative farms, three of them are also negative for PSV and PTV (Table 5 and Table S3). The highest prevalence of PSV was found in a farm in Füzesgyarmat (Farm ID: S157, 8/15 positive, 53.3%) while most of the PTV and PoAstV-3 positive samples were identified in Kaba (S151, 7/11 PTV positive, 63.6%) and Biharnagybajom (S153, 6/15 PoAstV-3 positive, 40.0%), respectively (Table 5, Figure 1). There were *n* = 5 farms where all three viruses were present, including the aforementioned farms at Kaba (S151) and Füzesgyarmat (S157) where the highest positivity rates of PSV and PTV were found. All three investigated viruses were found in relatively low prevalence (<30% prevalence, mostly 1–3 positive sample/farm) in most of the farms (66.7–75%) and only PTV was found in high prevalence (>60%) in a single farm (Table 5).

In addition to nasal swabs, CNS samples (brain stem, cerebellum and spinal cord) from paraplegic pigs with unknown etiology were also tested by our novel RT-qPCR assay, but none of the samples showed positivity with Cq values < 36.5 (Table S4).

## 4. Discussion

In this study, a novel, highly sensitive one-step triplex RT-qPCR assay was introduced, capable of the simultaneous detection of the selected neurotropic RNA viruses, i.e., porcine tescho-, and sapeloviruses of family *Picornaviridae,* and type 3 porcine astroviruses of family *Astroviridae*. These viruses could cause severe CNS infections with high mortality rate and—according to recent studies—are considered to be (re-)emerging pathogens of swine [1,2,3,4,5,6,7,8]. The fluorophore-tagged hydrolysis-probe-based assays were designed to either separately (singleplex) or simultaneously (triplex) detect all known types of species *Teschovirus A*, *B* and *Sapelovirus A* as well as specifically PoAstV-3 from various sample types including faecal, nasal or CNS samples of swine. The developed singleplex/triplex assays were characterized using in vitro generated PSV, PTV and PoAstV-3 RNA standards, and its clinical performances were compared to previously used RT-PCR screening methods. Moreover, the assay was successfully used in a large-scale epidemiological investigation on nasal swab samples.

The primers and probes of the assays targeted different, highly conserved genome parts (5′UTR of PTV, 3D-RdRp of PSV and ORF1ab-ORF2 junction of PoAstV-3) of the viruses of interest, which were identified using the separate alignments of currently available sequences. Compared to the numbers of available PTV (*n* = 128) and PSV (*n* = 105) sequences used for final alignments, only 18 complete or partial ORF1ab-ORF2 PoAstV-3 sequences were available for alignment. Due to the low number of known PoAstV-3 sequences used for primer–probe design, the clinical sensitivity of the developed PoAstV-3 might need further investigation as new virus sequences will be available. Indeed, during the preparation of the manuscript, 21 additional complete PoAstV-3 genome sequences have been published by Matias Ferreyra and co-workers from the USA [33], including 6 strains that are most likely undetectable by our PoAstV-3 assay due to an A/G mismatch located at the 3rd position at the 3′ end of the reverse primer (data not shown).

The assays were designed to produce PCR products in the sequenceable size range between 100 and 200 bp. Sequencing of the products could be used for verification of the RT-qPCR results as well as for sequence-specific primer design in case of the need for further sequence determination reactions (e.g., for genotyping). The generated sequences from the RT-qPCR assays are not suitable for genotype determination due to the short size and the conserved nature of the amplified genomic parts independent from the genotype. Note that the determined PSV, PTV and PoAstV-3 sequences from the RT-qPCR assays share 90–99%, 97–100%, 95–100% pairwise nt identity to each other, respectively (data not shown). The applied RT-qPCR protocol for singleplex/triplex assays was optimized for LightCycler^®^ Multiplex RNA Virus Master One-step RT-qPCR Master Mix (Roche), and all tests were run on a white plate or white 8-well-strips on a CFX96 Touch Real-Time PCR Detection System (Bio-Rad Laboratories, Inc., Hercules, CA, USA). Any changes in the type of master mix and/or the elements of the reaction setup could influence the performance of the assay and should be re-optimized accordingly.

During the analytical sensitivity and specificity tests of virus-specific singleplex and triplex assays, the slope (should be close to −3.322), correlation coefficient (R^2^, should be >0.980) and reaction efficiency (E, should be around 100%) [34] values were found in an acceptable range (Figure 4A–D), indicating the optimal performances of both assay types.

The lower limits of detection (a target concentration which was detectable in >95% of the repeats) [35] in singleplex and triplex assays were found to be 100 copies/reaction, but true amplification signals with higher Cq values were measured in lower concentrations (i.e., 10 copies/reaction) as well, although with variable (but <95%) detection rates (Table 2). Based on the distribution of Cq values measured at the lowest tested concentration (10 copies/reaction), the cut-off Cq was uniformly set to 36.5 of all three targets.

The diagnostic sensitivities of the RT-qPCR assays were compared to previously used RT-PCR-based screening tests on archived RNA samples which contained known PSV, PTV and PoAstV-3 positive and negative samples. The primers of both assay types (RT-PCR and RT-qPCR) were targeting the same genomic regions of the viruses. Both the calculated diagnostic sensitivity values of RT-qPCR assays and concordances of the results are less than 100%, but the sequencing of the PCR products of ambiguous samples causing the discrepancies (either RT-qPCR +/RT-PCR − or RT-qPCR −/RT-PCR +) indicate higher specificity of RT-qPCR assays than the RT-PCR tests used for comparisons (Table 4). The total number of false-positive samples is *n* = 3 (*n* = 2 PSV, *n* = 1 PTV and *n* = 0 PoAstV-3) of RT-qPCR compared to *n* = 11 (*n* = 3 PSV, *n* = 3 PTV and *n* = 5 PoAstV-3) of RT-PCR screening tests. Furthermore, the RT-qPCR assay showed consequent negative results (no determinable Cq or Cq > 36.5) on *n* = 21 different CNS samples. These results suggest the overall higher reliability (adequate sensitivity with high specificity) of our RT-qPCR assays in diagnostic cases of various types of samples.

Various tissues, including different CNS samples from PoAstV-3-infected paraplegic pigs (animal IDs: GD1–5, Table S2), which were described in our previous study [5], were also part of the analyzed archived RNA samples. Similar to our previous results, PoAstV-3 was detectable by RT-qPCR in the CNS samples as well as in tonsils, blood and nasal mucosa of paraplegic pigs (Table S2) [5]. Interestingly, PoAstV-3 was detectable by RT-qPCR in a single enteric sample (ileum) which was previously negative by PoAstV-3 RT-PCR screening assay (Table S2), suggesting the possibility of low-level faecal shedding of the virus during the acute paraplegia. PSV was detectable in the intestine and various extra-intestinal organs including mesenteric lymph nodes, liver, spleen, kidney and urine samples of PoAstV-3-infected paraplegic pigs (Table S2). Additionally, PTV was also present extra-intestinally in mesenteric lymph nodes, tonsils and nasal mucosa (Table S2). These results indicate disseminated PoAstV-3, PTV and PSV co-infections as well as PoAstV-3 neuro infections in these newly weaned paraplegic pigs at the time of acute CNS-related symptoms [5]. The triple disseminated infections could be responsible for the severity of the observed disease and could also support the suspected decreased immune status of these animals [5]. Although disseminated infections of PTVs, PSVs and PoAstV-3s with various manifested symptoms have also been demonstrated before [2,3,5,13,36], triple-infections have not been reported yet. All three enteric viruses were previously reported to be present in nasal samples to various extents [5,10,14,16], therefore, to gather additional data about the nasal presence of these viruses, multiple nasal swab samples were collected from approximately 3-week-old asymptomatic swine from 28 different farms in Hungary and a large-scale epidemiological investigation was conducted using our sensitive RT-qPCR assays.

From the three investigated viruses, PoAstV-3 was found to be overall the most prevalent (17.8% positivity) followed by PTV and PSV, which were detected in a much lower number (9.9% and 7.8% positivity) of the investigated nasal swab samples. Additional information is available only from the prevalence of PoAstV-3 in nasal swabs from swine of various ages, where the highest detection rate (30% positivity) was found in 3-week-old animals [16]. The Cq values of nasal samples are consistently higher (>28.11 of PSV, >28.27 of PTV and >31.67 of PoAstV-3) compared to the values measured in enteric samples (>18.55 of PSV, >18.39 of PTV and >24.41 of PoAstV-3). Similarly, considerably higher Cq values of PoAstV-3 and PTV were reported in nasal swabs than those measured in faeces [14,16]. To our knowledge, similar RT-qPCR-based comparative analyses of PSV shedding in nasal sand enteric samples have not been done before.

The prevalence of PoAstV-3 was also the highest among the investigated farms (85% of the farms are PoAstV-3 positive), while PTV and PSV were detectable in a much lower number of herds (57% and 32% of the farms were positive, respectively). These results could either indicate the low level of virus secretion throughout the nose or the passive, non-replicating presence of these viruses in the nasal cavity. The high Cq values and the sporadic presence of these viruses in the nasal samples (only 1–3 positive sample/farm) of most of the positive farms (66.7–75%) could support—at least in some cases/viruses—the environmental origin of these viruses in the nose, as suspected by others [16]. Nevertheless, the presence of PSV, PTV and PoAstV-3 in nasal samples strongly indicate the circulation of these viruses in the positive farms. Based on these results, all three neurotropic viruses, especially PoAstV-3, are proven to be widespread and endemically present in most of the highly prolific, industrial-type swine farms across Hungary.

## 5. Conclusions

The one-step triplex RT-qPCR assay introduced in this study was proven to be capable of the sensitive and specific detection of sapelo-, tescho-, and type 3 porcine astroviruses in various sample types with diverse nucleic acid environments (i.e., the nucleic acid diversity of faecal, nasal and tissue samples are substantially different). Based on the results of specificity and sensitivity tests, this RT-qPCR assay could be useful in the accurate diagnosis of PSV, PTV and/or PoAstV-3 infection(s) in swine. Furthermore, the results of RT-qPCR-based large-scale epidemiological investigations demonstrate the widespread presence of these neurotropic viruses in Hungarian swine herds, which could raise veterinary concerns, especially in those farms where all three neurotropic viruses are co-circulating. Specific and sensitive assays, similar to those introduced here, are required for diagnostic testing of these viruses in swine, especially in the cases with CNS-related symptoms, including leg weakness, paraplegia and/or where paralysis is present.

## Figures and Tables

**Table 1:** List and characteristics of oligonucleotide primers and probes (with PR acronym) used for singleplex/triplex RT-qPCR assays in this study. F: forward, R: reverse, Tm: melting temperature. 6-FAM: 6-Carboxyfluorescein; SUN: a fluorophore with similar spectral characteristics to HEX and VIC dyes. Cy5: Cyanine 5/IABkFQ/3’ Iowa Black^®^ FQ quencher; /IAbRQSp/3’ Iowa Black^®^ RQ quencher.

Target Virus (Genome Region)	Oligonucleotide ID	Sequence (5′–3′)	Length (nt)	Tm (°C)	PCR Product Size (bp)
Sapelovirus A (3D-RdRp)	pan-PSV-3D-qPCR-R	CTC CTC ACC ATT GTG CCA TGC	21	65	132
	pan-PSV-3D-qPCR-F	TAC CCC TTC CTG TGT CAT CCT	21	64	
	pan-PSV-3D-qPCR-PR	/6-FAM/CAA TTA GAT GGA CCA AGA ATG CCA G/IABkFQ/	25	65	
Teschovirus A&B (5′UTR)	pan-PTV-5UTR-qPCR-R	TGA CTA TAC AAA GTA CAG ACG GCC	24	64	172
	pan-PTV-5UTR-qPCR-F	GTA CAG AAG AGC AAG TAC TCC TGA	24	64	
	pan-PTV-5UTR-qPCR-PR	/SUN/GGA GTT CCT GTA TGG GAA TGC A/IABkFQ/	22	65	
Porcine astrovirus type 3	PoAstV3-CAP-qPCR-R	TCC TTG GCA ACC TCC TTA GC	20	64	178
(ORF1b-ORF2 junction)	PoAstV3-RdRp-qPCR-F	GGT GGC CTT GGA GGT TGT T	19	64	
	PoAstV3-qPCR-PR	/Cy5/CTG ATG AGA TGC TTG ACC GGC/IAbRQSp/	21	65	

**Table 2:** Summary of the results of lower limit of detection analyses. Cq: quantification cycle, SD: standard deviation, PSV: porcine sapelovirus, PTV: porcine teschovirus, PoAstV-3: porcine astrovirus type 3.

Copies/Reaction	Mean Cq (SD)						Detected/Tested (%)					
	Singleplex			Triplex			Singleplex			Triplex		
	PSV	PTV	PoAstV-3	PSV	PTV	PoAstV-3	PSV	PTV	PoAstV-3	PSV	PTV	PoAstV-3
10	35.92 (0.63)	34.45 (1.31)	36.21 (1.30)	34.82 (1.20)	35.75 (0.95)	34.56 (0.63)	9/11 (82)	4/11 (36)	5/11 (46)	7/11 (64)	5/11 (46)	6/11 (55)
100	32.61 (0.50)	31.50 (1.04)	31.87 (0.91)	31.06 (0.59)	31.23 (0.53)	30.87 (0.31)	11/11 (100)	11/11 (100)	11/11 (100)	11/11 (100)	11/11 (100)	11/11 (100)
1000	29.02 (0.56)	28.33 (0.25)	28.31 (0.83)	28.25 (0.36)	28.29 (0.35)	28.22 (0.24)	11/11 (100)	11/11 (100)	11/11 (100)	11/11 (100)	11/11 (100)	11/11 (100)
10,000	25.77 (0.43)	25.48 (0.31)	25.11 (0.36)	25.88 (0.22)	25.86 (0.23)	25.63 (0.41)	6/6 (100)	6/6 (100)	6/6 (100)	6/6 (100)	6/6 (100)	6/6 (100)

**Table 3:** Summary of the results of intra/inter assay coefficients of variation (CV%). PSV: porcine sapelovirus, PTV: porcine teschovirus, PoAstV-3: porcine astrovirus type 3. 1 × 10^2^, 1 × 10^3^: copies/reaction.

	Intra-Assay Variation (CV%)				Inter-Assay Variation (CV%)			
	Singleplex		Triplex		Singleplex		Triplex	
Virus	1 × 10^2^	1 × 10^3^	1 × 10^2^	1 × 10^3^	1 × 10^2^	1 × 10^3^	1 × 10^2^	1 × 10^3^
PSV	0.05–1.55	0.12–1.87	0.03–1.75	0.13–0.32	1.53	1.92	1.91	1.26
PTV	0.03–4.62	0.10–0.88	0.13–1.62	0.11–0.91	3.29	0.87	1.70	1.22
PoAstV-3	0.63–3.58	0.29–3.38	0.10–0.88	0.20–1.53	2.87	2.95	1.02	0.86

**Table 4:** Summary of the clinical performance of triplex RT-qPCR assay (tRT-qPCR) based on the comparison with previously published RT-PCR-based screening assays on *n* = 142 selected archived samples of swine. For the details of the comparisons, see Supplementary Table S2. Seq+: number of true-positive samples confirmed by Sanger-sequencing.

	Results of tRT-qPCR vs. RT-PCR (No. of Samples (Seq+))				Performance of tRT-qPCR Compared to RT-PCR		
Virus	+/+	+/−	−/+	−/−	Sensitivity (%)	Specificity (%)	Concordance (%)
PSV	57	7 (5)	6 (3)	72	95.59	97.30	90.85
PTV	42	5 (4)	7 (4)	88	92.59	98.88	91.55
PoAstV-3	18	2 (2)	7 (2)	115	91.67	100.00	93.66

**Table 5:** Positivity rates (no. of positive/no. of tested with percentages) of porcine sapelovirus (PSV), porcine teschovirus (PTV) and porcine astrovirus type 3 (PoAstV-3), including double and triple infections identified by RT-qPCR in *n* = 473 nasal swab samples of *n* = 28 different Hungarian swine farms. The highest prevalence values of PSV, PTV or PoAstV-3 are marked in bold. For detailed RT-qPCR results, see Table S3.

Farm ID	Location	PSV	PTV	PoAstV-3	PSV &PTV	PSV&	PTV&	Triple Infection
						PoAstV-3	PoAstV-3	
S105	Sajószöged	0/13	1/13 (7.7%)	4/13	0/13	0/13	1/13	0/13
				(30.8%)			(7.7%)	
S126	Szarvas	0/13	0/13	0/13	0/13	0/13	0/13	0/13
S127	Cibakháza	0/10	1/10	3/10	0/10	0/10	0/10	0/10
			(10.0%)	(30.0%)				
S128	Hajdúnánás	0/12	0/12	4/12	0/12	0/12	0/12	0/12
				(33.3%)				
S150	Hajdószoboszló	0/15	7/15	3/15	0/15	0/15	1/15	0/15
			(46.6%)	(20.0%)			(6.6%)	
S151	Kaba	2/11	**7/11**	3/11	0/11	0/11	2/11	1/11
		(18.1%)	**(63.6%)**	(27.3%)			(18.2%)	(9.1%)
S152	Karcag	0/15	0/15	2/15	0/15	0/15	0/15	0/15
				(13.3%)				
S153	Biharnagybajom	0/15	0/15	**6/15**	0/15	0/15	0/15	0/15
				**(40.0%)**				
S154	Nádudvar	0/15	3/15	3/15	0/15	0/15	1/15	0/15
			(20.0%)	(20.0%)			(6.7%)	
S155	Nádudvar	0/14	1/14	4/14	0/14	0/14	0/14	0/14
			(7.1%)	(28.6%)				
S156	Püspökladány	0/15	0/15	2/15	0/15	0/15	0/15	0/15
				(13.3%)				
S157	Füzesgyarmat	**8/15**	6/15	3/15	3/15	1/15	2/15	0/15
		**(53.3%)**	(40.0%)	(20.0%)	(20.0%)	(6.7%)	(13.3%)	
S159	Kocs	0/14	0/14	3/14	0/14	0/14	0/14	0/14
				(21.4%)				
S162	Fadd	4/9	2/9	3/9	1/9	1/9	0/9	1/9
		(44.4%)	(22.2%)	(33.3%)	(11.1%)	(11.1%)		(11.1%)
S163	Dunaszekcső	3/10	0/10	0/10	0/10	0/10	0/10	0/10
		(30.0%)						
S164	Dávod	2/15	2/15	5/15	1/15	1/15	1/15	0/15
		(13.3%)	(13.3%)	(33.3%)	(6.7%)	(6.7%)	(6.7%)	
S165	Óalmás	0/15	7/15	5/15	0/15	0/15	2/15	0/15
			(46.7%)	(33.3%)			(13.3%)	
S166	Somberek	0/15	0/15	0/15	0/15	0/15	0/15	0/15
S167	Mohács	0/14	2/14	3/14	0/14	0/14	0/14	0/14
			(14.3%)	(21.4%)				
S168	Újpetre	0/13	0/13	2/13	0/13	0/13	0/13	0/13
				(15.4%)				
S169	Töttös	0/15	2/15	2/15	0/15	0/15	1/15	0/15
			(13.3%)	(13.3%)			(6.7%)	
S216	Szarvas	0/9	0/9	0/9	0/9	0/9	0/9	0/9
S234	Dávod	2/30	1/30	3/30	0/30	0/30	1/30	0/30
		(6.7%)	(3.3%)	(10.0%)			(3.3%)	
S240	Töttös	1/30	0/30	3/30	0/30	0/30	0/30	0/30
		(3.3%)		(10.0%)				
SZV	Szigetvár	7/29	0/29	2/29	0/29	0/29	0/29	0/29
		(24.1%)		(6.9%)				
BSZ	Bácsalmás	0/42	2/42	7/42	0/42	0/42	1/42	0/42
			(4.8%)	(16.7%)			(2.4%)	
DSZ	Dunaszekcső	0/31	1/31	6/31	0/31	0/31	0/31	0/31
			(3.2%)	(19.3%)				
GD	Gádoros	9/19	2/19	3/19	2/19	3/19	0/19	0/19
		(47.4%)	(10.5%)	(15.8%)	(10.5%)	(15.8%)		

## Data Availability

Data is contained within the article or supplementary material.

## Supplementary Materials

The following are available online at https://www.mdpi.com/article/10.3390/v14030513/s1. Technical appendix: Detailed description of the production process of PSV, PTV and PoAstV-3 RNA standards. Figure S1: Bean plot of Cq values of 1 × 10^1^ copies/reaction of RNA standards measured in porcine sapelovirus (PSV), porcine teschovirus (PTV) and porcine astrovirus type 3 (PoAstV-3) RT-qPCR assays. Table S1: List and characteristics of oligonucleotide primers used in this study. Table S2: Comparison of the clinical performances of triplex RT-qPCR (tRT-qPCR) and classical RT-PCR assays in *n* = 142 archived RNA samples of swine (*n* = 100 faecal/rectal swabs, *n* = 1 urine and *n* = 41 tissue specimens). Table S3: Results of porcine sapelovirus (PSV), porcine teschovirus (PTV) and porcine astrovirus type 3 (PoAstV3) RT-qPCR assays of *n* = 473 nasal swabs collected in *n* = 28 different Hungarian swine farms. Table S4: Results of porcine sapelovirus (PSV), porcine teschovirus (PTV) and porcine astrovirus type 3 (PoAstV3) RT-qPCR assays of *n* = 21 samples from various sites of the central nervous system of *n* = 8 paraplegic pig with unknown etiology.
