# Supplementary material for: Development and Large-Scale Testing of a Novel One-Step Triplex RT-qPCR Assay for Simultaneous Detection of “Neurotropic” Porcine Sapeloviruses, Teschoviruses (Picornaviridae) and Type 3 Porcine Astroviruses (Astroviridae) in Various Samples including Nasal Swabs
Source: Viruses. 2022 Mar 2;14(3):513. doi: 10.3390/v14030513 (PMC8952109; doi:10.3390/v14030513)
Supplement: Supplementary file 1 [file viruses-14-00513-s001.zip › Figure S1 - viruses-1487148.pdf]

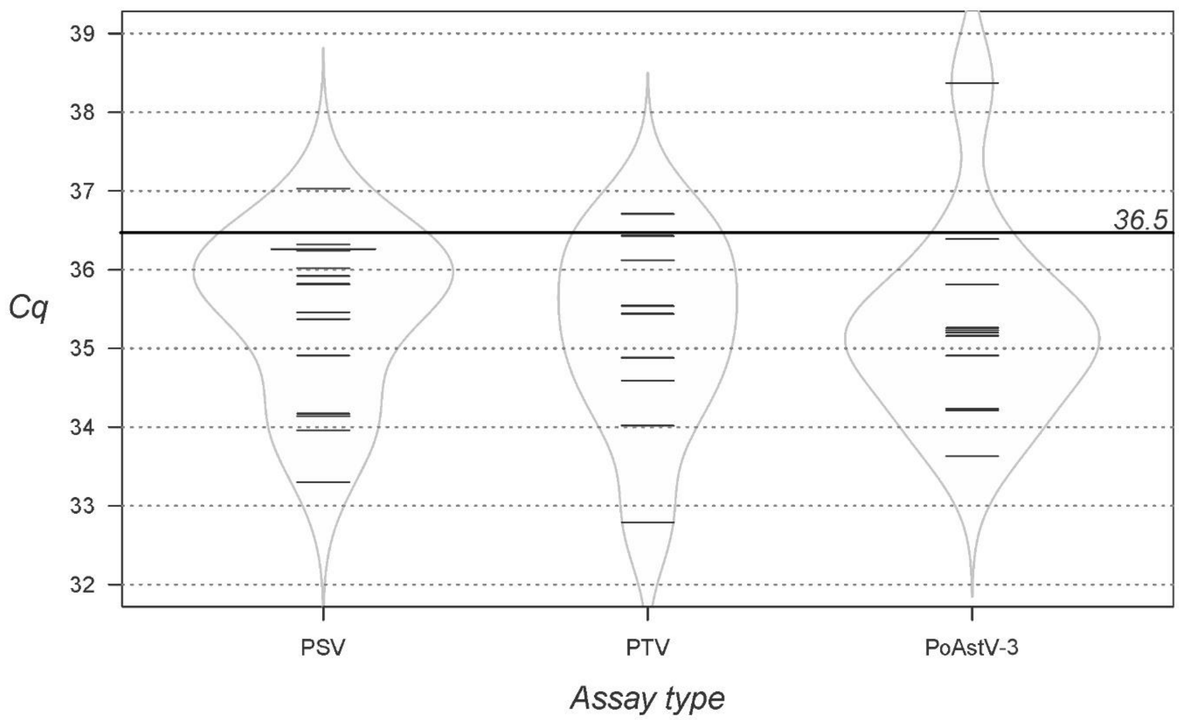

Figure S1: Bean plot of Cq values of 1 × 10^1 copies/reaction of RNA standards measured in porcine sapelovirus (PSV), porcine teschovirus (PTV) and porcine astrovirus type 3 (PoAstV-3) RT-qPCR assays. Grey lines represent individual data points; polygons represent the estimated density of the data. Horizontal black line indicates the cut-off Cq value which was arbitrarily set to 36.5. The cut-off Cq threshold was set to be located between the highest and second highest Cq values in case of all three viruses.
