# Supplementary material for: Development and Large-Scale Testing of a Novel One-Step Triplex RT-qPCR Assay for Simultaneous Detection of “Neurotropic” Porcine Sapeloviruses, Teschoviruses (Picornaviridae) and Type 3 Porcine Astroviruses (Astroviridae) in Various Samples including Nasal Swabs
Source: Viruses. 2022 Mar 2;14(3):513. doi: 10.3390/v14030513 (PMC8952109; doi:10.3390/v14030513)
Supplement: Supplementary file 1 [file viruses-14-00513-s001.zip › Supplemetary Table S1 - viruses-1487148.pdf]

Target virus (genome region)	Reaction type	Oligonucleotide ID	Sequence (5' - 3')	Length (nt)	Tm (°C)	PCR product size (bp)	Reference
Sapelovirus A (3D-RdRp)	RT-PCR (standard)	PSV2-qPCR-Contr-R	AGC CCT ACC TAC TGG TAC TGA T	22	64		this study
		PSV2-qPCR-Contr-F	GGC TGC TCC GGA ACT AGT GTT	21	66	470	this study
	RT-PCR (screen)	PSV-3Dscreen-6595-R	CCA AAR GTY TGC TTC ATC ATT AT	23	59	387	[10]
		PSV-3Dscreen-6209-F	TGT TGA TGC YAC MCT TCC AGA	21	61		[10]
Teschovirus A&B (5'UTR)	RT-PCR (standard)	PTV-qPCR-Contr-R	CCA TGA GTC ATA CTC AAC TTC TGC	24	63		this study
		PTV-qPCR-Contr-F	CCA GGT ATT GCA CCA ATG GT	20	63	406	this study
	RT-PCR (screen)	Teschovirus-5UTR-Rgen	CCA GCC GCG ACC CTG TCA GGC A	22	74	321	[21]
		Teschovirus-5UTR-Fgen	AGT TTT GGA TTA TCT TGT GCC C	22	61		[21]
Porcine astrovirus type 3	RT-PCR (standard)	PoAstV3-qPCR-Contr-R	GCC TGA TTT CTC TTC TCC TCC T	22	62		this study
(ORF1b-ORF2 junction)		PoAstV3-qPCR-Contr-F	TAC AGA TGA GGA ACT CGA GGC A	22	63	591	this study
Porcine astrovirus type 3 (ORF1b)	RT-PCR (screen)	PoAsV3-Screen-R	AAC CYT CTC CAC ATA ATT GGC	21	60	356	[5]
		PoAsV3-Screen-F	GAA GTG TTT ATG CAC ATC AAG A	22	59		[5]

Table S1: List and characteristics of oligonucleotide primers used in this study. F: forward, R: reverse, Tm: melting temperature, RT-PCR (screen): primers used for screening RT-PCR reactions. RT-PCR (standard): primers used for the generation of double-stranded DNA templates used for *in vitro* production of virus-specific RNA standards.
