# Supplementary material for: Development and Large-Scale Testing of a Novel One-Step Triplex RT-qPCR Assay for Simultaneous Detection of “Neurotropic” Porcine Sapeloviruses, Teschoviruses (Picornaviridae) and Type 3 Porcine Astroviruses (Astroviridae) in Various Samples including Nasal Swabs
Source: Viruses. 2022 Mar 2;14(3):513. doi: 10.3390/v14030513 (PMC8952109; doi:10.3390/v14030513)
Supplement: Supplementary file 1 [file viruses-14-00513-s001.zip › Tables S2-S3-S4 - viruses-1487148.pdf]

Farm ID	Location	Date (sampling)	Sample type	Sample ID	Age	Health status	PSV (RT-PCR)	PSV (tRT-qPCR)	PTV (RT-PCR)	PTV (tRT-qPCR)	PoAstV-3 (RT-PCR)	PoAstV-3 (tRT-qPCR)	References of the samples
Farm A	Városföld	08/12/ 2016	Rectal swab	A1	≈35 days	AS	+	21.39	-	-	-	-	[10]
	Városföld	08/12/ 2016	Rectal swab	A2	≈35 days	AS	+	23.14	+	23.27	-	-	[10]
	Városföld	08/12/ 2016	Rectal swab	A3	≈35 days	AS	+	18.55	+	21.15	-	-	[10]
	Városföld	08/12/ 2016	Rectal swab	A4	≈35 days	AS	-	-	-	-	-	-	[10]
	Városföld	08/12/ 2016	Rectal swab	A5	≈35 days	AS	+	23.20	-	-	-	-	[10]
Farm B	Katymár	08/18/ 2016	Rectal swab	B1	≈35 days	AS	+	20.43	-	-	-	-	[10]
	Katymár	08/18/ 2016	Rectal swab	B2	≈35 days	AS	+	22.66	+	26.16	-	-	[10]
	Katymár	08/18/ 2016	Rectal swab	B3	≈35 days	AS	-	-	-	-	-	-	[10]
	Katymár	08/18/ 2016	Rectal swab	B4	≈35 days	AS	+	20.61	+	22.53	-	-	[10]
	Katymár	08/18/ 2016	Rectal swab	B5	≈35 days	AS	+	26.02	-	-	-	-	[10]
Farm C	Bácsalmás	08/18/ 2016	Rectal swab	C1	≈35 days	AS	+	19.57	+	26.01	-	-	[10]
	Bácsalmás	08/18/ 2016	Rectal swab	C2	≈35 days	AS	+	24.93	+	24.33	-	-	[10]
	Bácsalmás	08/18/ 2016	Rectal swab	C3	≈35 days	AS	+	20.86	+	26.43	+ (Seq-)	-	[10]
	Bácsalmás	08/18/ 2016	Rectal swab	C4	≈35 days	AS	+	22.07	+	27.54	-	-	[10]
	Bácsalmás	08/18/ 2016	Rectal swab	C5	≈35 days	AS	+	19.60	-	-	-	-	[10]
Farm D	Nyíribrony	08/10/ 2016	Rectal swab	D1	≈35 days	AS	-	-	+	24.51	-	-	[10]
	Nyíribrony	08/10/ 2016	Rectal swab	D2	≈35 days	AS	-	-	-	26.65 (Seq-)	-	-	[10]
	Nyíribrony	08/10/ 2016	Rectal swab	D3	≈35 days	AS	-	-	-	-	-	-	[10]
	Nyíribrony	08/10/ 2016	Rectal swab	D4	≈35 days	AS	-	30.00 (Seq+)	-	-	-	-	[10]
	Nyíribrony	08/10/ 2016	Rectal swab	D5	≈35 days	AS	-	-	-	26.92 (Seq+)	-	-	[10]
Farm TM	Tázlár	01/2013	Faeces	TKo-1	≈ 11-12 mo.	AS	-	-	-	-	-	-	[10]
	Tázlár	01/2013	Faeces	TKo-2	≈ 11-12 mo.	AS	-	-	-	-	-	-	[10]
	Tázlár	01/2013	Faeces	TKo-3	≈ 11-12 mo.	AS	-	29.57 (Seq+)	-	-	-	-	[10]
	Tázlár	01/2013	Faeces	TM-1121	7-21 days	PP	+	20.14	-	-	-	-	[10]
	Tázlár	01/2013	Faeces	TM-1130	7-21 days	PP	-	-	-	-	-	-	[10]
	Tázlár	01/2013	Faeces	TM-1205	7-21 days	PP	-	20.54 (Seq+)	-	-	+	24.59	[10]
Farm NSZ	Nagyszokoly	01/23/ 2013	Faeces	NSzK1	≈ 11-12 mo.	PP	-	-	-	-	-	-	[10]
Farm KEV	Kevermes	09/05/ 2013	Faeces	KEV-1	≈ 4.5 mo.	PP	-	-	-	-	-	-	[10]
	Kevermes	09/05/ 2013	Faeces	KEV-2	≈ 4.5 mo.	PP	-	-	-	-	-	-	[10]
	Kevermes	09/05/ 2013	Faeces	KEV-3	≈ 4.5 mo.	PP	-	-	+	27.61	-	-	[10]
Farm ZS	Zsana	04/14/ 2013	Faeces	Zsana-1	≈70 days	PP	+	23.83	-	-	-	-	[10]
	Zsana	04/14/ 2013	Faeces	Zsana-2	≈70 days	PP	+	23.25	-	28.08 (Seq+)	-	-	[10]
	Zsana	04/14/ 2013	Faeces	Zsana-3	≈70 days	PP	+	23.52	-	-	-	-	[10]
Farm BUV	Balmazújváros	04/24/ 2013	Faeces	BUV-1	21-35 days	PP	+	23.01	+	24.32	+ (Seq-)	-	[10]
	Balmazújváros	04/24/ 2013	Faeces	BUV-2	21-35 days	PP	+	28.10	+	26.40	-	-	[10]
Farm SZ	Székelyszabar	01/2013	Faeces	SZ1K	≈ 11-12 mo.	AS	-	-	-	-	-	-	[10]
	Székelyszabar	01/2013	Faeces	SZ2K	≈ 11-12 mo.	AS	-	-	+	24.03	-	-	[10]
	Székelyszabar	01/2013	Faeces	SZK3	≈ 11-12 mo.	AS	-	-	+	24.01	-	-	[10]
	Székelyszabar	01/2013	Faeces	SZK4	≈ 11-12 mo.	AS	+ (Seq+)	-	+	27.34	-	-	[10]
	Székelyszabar	01/2013	Faeces	SZ1M	7-21 days	PP	+	23.61	+	19.69	-	-	[10]
	Székelyszabar	01/2013	Faeces	SZ2M	7-21 days	PP	-	-	+	21.34	-	-	[10]
	Székelyszabar	01/2013	Faeces	SZ3M	7-21 days	PP	-	-	-	-	-	-	[10]
	Székelyszabar	01/2013	Faeces	SZ4M	7-21 days	PP	+	21.25	-	-	-	-	[10]
Farm EF	Egyházasfalu	08/17/ 2016	Faeces	EF1	≈ 3 mo.	AS	-	-	+	23.31	-	-	[10]
	Egyházasfalu	08/17/ 2016	Faeces	EF2	≈ 3 mo.	AS	+	26.60	+	22.47	-	-	[10]

	Egyházasfalu	08/17/ 2016	Faeces	EF3	≈ 3 mo.	AS	+	27.60	+	26.90	-	-	[10]
	Egyházasfalu	08/17/ 2016	Faeces	EF4	≈ 3 mo.	AS	+	26.62	-	-	-	-	[10]
	Egyházasfalu	08/17/ 2016	Faeces	EF5	≈ 3 mo.	AS	+ (Seq+)	-	-	-	-	-	[10]
	Egyházasfalu	08/17/ 2016	Faeces	EF6	≈ 3 mo.	AS	+	26.03	+	25.22	-	-	[10]
	Egyházasfalu	08/17/ 2016	Faeces	EF7	≈ 3 mo.	AS	+	25.22	+	26.07	-	-	[10]
	Egyházasfalu	08/17/ 2016	Faeces	EF8	≈ 3 mo.	AS	+	19.25	+	24.69	-	-	[10]
	Egyházasfalu	08/17/ 2016	Faeces	EF9	≈ 3 mo.	AS	+	25.40	+	26.29	-	-	[10]
	Egyházasfalu	08/17/ 2016	Faeces	EF10	≈ 3 mo.	AS	+	29.59	+	23.20	-	-	[10]
Farm EBES	Ebes	01/11/2008	Faeces	K1	≈ 4 mo.	AS	+	29.55	-	-	-	-	[10]
	Ebes	01/11/2008	Faeces	K2	≈ 4 mo.	AS	+	27.05	+	27.90	-	-	[10]
	Ebes	01/11/2008	Faeces	K3	≈ 4 mo.	AS	-	-	-	-	-	-	[10]
	Ebes	01/11/2008	Faeces	K4	≈ 4 mo.	AS	-	31.09 (Seq-)	-	-	-	-	[10]
	Ebes	01/11/2008	Faeces	K5	≈ 4 mo.	AS	+	30.31	-	-	-	-	[10]
	Ebes	01/11/2008	Faeces	K7	≈ 4 mo.	AS	+	28.12	+	26.47	-	-	[10]
	Ebes	01/11/2008	Faeces	K10	≈ 4 mo.	AS	+	30.15	+	29.29	-	-	[10]
	Ebes	01/11/2008	Faeces	K21	≈10 days	AS	-	-	-	-	-	-	[10]
	Ebes	01/11/2008	Faeces	K22	≈10 days	AS	-	-	-	-	-	-	[10]
	Ebes	01/11/2008	Faeces	K23	≈10 days	AS	-	-	-	-	-	-	[10]
	Ebes	01/11/2008	Faeces	K24	≈10 days	AS	-	-	-	-	-	-	[10]
	Ebes	01/11/2008	Faeces	K25	≈10 days	AS	-	-	+ (Seq+)	-	-	-	[10]
	Ebes	01/11/2008	Faeces	K26	≈10 days	AS	-	-	-	-	-	-	[10]
	Ebes	01/11/2008	Faeces	K27	≈10 days	AS	-	-	-	-	+ (Seq-)	-	[10]
	Ebes	01/11/2008	Faeces	K28	≈10 days	AS	-	-	-	-	-	-	[10]
	Ebes	01/11/2008	Faeces	K29	≈10 days	AS	-	-	-	-	-	-	[10]
	Ebes	01/11/2008	Faeces	K30	≈10 days	AS	+ (Seq+)	-	-	-	-	24.41 (Seq+)	[10]
	Ebes	01/11/2008	Faeces	K31	≈ 3 mo.	AS	-	-	-	-	-	-	[10]
	Ebes	01/11/2008	Faeces	K32	≈ 3 mo.	AS	-	-	-	-	-	-	[10]
	Ebes	01/11/2008	Faeces	K33	≈ 3 mo.	AS	-	-	-	-	-	-	[10]
	Ebes	01/11/2008	Faeces	K34	≈ 3 mo.	AS	-	-	-	-	-	-	[10]
	Ebes	01/11/2008	Faeces	K35	≈ 3 mo.	AS	-	-	-	-	-	-	[10]
	Ebes	01/11/2008	Faeces	K36	≈ 3 mo.	AS	-	-	-	-	-	-	[10]
Farm GD	Orosháza	09/15/ 2016	Rectal swab	GD_01	≈21-25 days	PP	+	28.63	-	-	-	-	[10]
	Orosháza	09/15/ 2016	Rectal swab	GD_02	≈21-25 days	PP	+	24.51	+ (Seq+)	-	-	-	[10]
	Orosháza	09/15/ 2016	Rectal swab	GD_03	≈21-25 days	PP	+	35.37	-	-	-	-	[10]
	Orosháza	09/15/ 2016	Rectal swab	GD_04	≈21-25 days	PP	-	-	-	-	+ (Seq-)	-	[10]
	Orosháza	09/15/ 2016	Rectal swab	GD_05	≈21-25 days	AS	+	30.03	-	32.10	-	-	[10]
	Orosháza	09/15/ 2016	Rectal swab	GD_06	≈21-25 days	AS	+	30.84	-	-	-	-	[10]
	Orosháza	09/15/ 2016	Rectal swab	GD_07	≈21-25 days	AS	+	23.20	-	-	-	-	[10]
	Orosháza	09/15/ 2016	Rectal swab	GD_09	≈21-25 days	AS	+	27.38	-	-	-	-	[10]
Farm WB-BF	Bőszénfa	04/22/2011	Faeces	WB2A	≈ 2 mo.	AS	-	-	+	23.34	-	-	[28]
	Bőszénfa	04/22/2011	Faeces	WB2B	≈ 2 mo.	AS	+	27.01	+	18.39	-	-	[28]
	Bőszénfa	04/22/2011	Faeces	WB2C	≈ 2 mo.	AS	+	24.74	+	21.32	-	-	[28]
	Bőszénfa	04/22/2011	Faeces	WB2D	≈ 2 mo.	AS	-	-	+	22.94	-	-	[28]
	Bőszénfa	04/22/2011	Faeces	WB2E	≈ 2 mo.	AS	-	-	-	28.16 (Seq+)	-	-	[28]
Farm WB-TF	Tótfalu	17/05/2011	Faeces	WB-TF-1	≈ 2 mo.	AS	+	24.89	-	-	-	-	[28]
	Tótfalu	17/05/2011	Faeces	WB-TF-2	≈ 2 mo.	AS	-	-	+ (Seq+)	-	-	-	[28]
	Tótfalu	17/05/2011	Faeces	WB-TF-3	≈ 2 mo.	AS	-	-	-	-	-	-	[28]
	Tótfalu	17/05/2011	Faeces	WB-TF-4	≈ 2 mo.	AS	+	27.43	-	-	-	-	[28]

	Tótfalu	17/05/2011	Faeces	WB-TF-5	≈ 2 mo.	AS	+	28.68	-	-	+ (Seq-)	-	[28]
	Tótfalu	17/05/2011	Faeces	WB-TF-6	≈ 2 mo.	AS	-	-	+	29.01	-	-	[28]
	Tótfalu	17/05/2011	Faeces	WB-TF-7	≈ 2 mo.	AS	-	-	-	-	-	-	[28]
	Tótfalu	17/05/2011	Faeces	WB-TF-8	≈ 2 mo.	AS	-	-	-	-	-	-	[28]
	Tótfalu	17/05/2011	Faeces	WB-TF-9	≈ 2 mo.	AS	-	30.56 (Seq+)	-	-	-	-	[28]
	Tótfalu	17/05/2011	Faeces	WB-TF-10	≈ 2 mo.	AS	+	23.16	+	21.61	-	-	[28]
	Tótfalu	17/05/2011	Faeces	WB-TF-11	≈ 2 mo.	AS	-	-	-	-	-	-	[28]
Farm GD	Orosháza	03/2016	Brain stem (BS)	GD-1-BS	≈21-25 days	PP	-	-	-	-	+	22.39	[5]
	Orosháza	03/2016	Brain stem (BS)	GD-2-BS	≈21-25 days	PP	-	-	-	-	-	-	[5]
	Orosháza	07/2016	Brain stem (BS)	GD-3-BS	≈21-25 days	PP	-	-	-	-	+	14.60	[5]
	Orosháza	07/2016	Brain stem (BS)	GD-4-BS	≈21-25 days	PP	+ (Seq-)	-	-	-	+	14.82	[5]
	Orosháza	11/2015	Brain stem (BS)	GD-5-BS	≈21-25 days	PP	-	-	-	-	+	16.58	[5]
	Orosháza	07/2016	Brain stem (BS)	GD-6-BS	≈35 days	AS	-	-	+ (Seq-)	-	-	-	[5]
	Orosháza	06/2017	Brain stem (BS)	GD-7-BS	≈25 days	AS	-	-	-	-	-	-	[5]
	Orosháza	06/2017	Brain stem (BS)	GD-8-BS	≈25 days	AS	-	-	-	-	-	-	[5]
	Orosháza	06/2017	Brain stem (BS)	GD-9-BS	≈25 days	AS	-	-	-	-	-	-	[5]
	Orosháza	06/2017	Brain stem (BS)	GD-10-BS	≈35 days	AS	-	-	-	-	-	-	[5]
	Orosháza	03/2016	Spinal cord - lumbar (SC-L)	GD-1-SC-L	≈21-25 days	PP	-	-	-	-	+	16.39	[5]
	Orosháza	07/2016	Spinal cord - cervical (SC-C)	GD-3-SC-C	≈21-25 days	PP	-	-	+ (Seq-)	-	+	17.66	[5]
	Orosháza	07/2016	Spinal cord - thoracic (SC-T)	GD-3-SC-T	≈21-25 days	PP	-	-	-	-	+	19.82	[5]
	Orosháza	07/2016	Spinal cord - lumbar (SC-L)	GD-3-SC-L	≈21-25 days	PP	+ (Seq-)	-	-	-	+	14.69	[5]
	Orosháza	07/2016	Spinal cord - lumbar (SC-L)	GD-4-SC-L	≈21-25 days	PP	-	-	-	-	+	17.81	[5]
	Orosháza	07/2016	Spinal cord - cervical (SC-C)	GD-4-SC-C	≈21-25 days	PP	-	-	-	-	+	18.15	[5]
	Orosháza	07/2016	Spinal cord - thoracic (SC-T)	GD-4-SC-T	≈21-25 days	PP	-	-	-	-	+	18.26	[5]
	Orosháza	11/2015	Spinal cord - lumbar (SC-L)	GD-5-SC-L	≈21-25 days	PP	-	-	+ (Seq-)	-	+	21.55	[5]
	Orosháza	07/2016	Spinal cord - lumbar (SC-L)	GD-6-SC-L	≈35 days	AS	-	-	-	-	-	-	[5]
	Orosháza	07/2016	Intestine - ileum (ILE)	GD-3-ILE	≈21-25 days	PP	+	30.97	+	23.37	-	-	[5]
	Orosháza	07/2016	Intestine - ileum (ILE)	GD-4-ILE	≈21-25 days	PP	+	31.69	+	25.72	-	-	[5]
	Orosháza	07/2016	Intestine - ileum (ILE)	GD-6-ILE	≈35 days	AS	-	-	+	23.21	-	-	[5]
	Orosháza	11/2015	Intestine - ileum (ILE)	GD-5-ILE	≈21-25 days	PP	-	-	+ (Seq+)	-	-	29.44 (Seq+)	[5]
	Orosháza	07/2016	Lung	GD-3-LUNG	≈21-25 days	PP	-	-	-	-	+ (Seq+)	-	[5]
	Orosháza	07/2016	Nasal mucosa (NM)	GD-3-NM	≈21-25 days	PP	-	-	-	-	+	22.75	[5]
	Orosháza	07/2016	Nasal mucosa (NM)	GD-4-NM	≈21-25 days	PP	-	-	+	27.98	+	22.22	[5]
	Orosháza	07/2016	Nasal mucosa (NM)	GD-6-NM	≈35 days	AS	-	-	-	-	-	-	[5]
	Orosháza	03/2016	Tonsil (TON)	GD-2-TON	≈21-25 days	PP	-	-	+	31.73	-	-	[5]
	Orosháza	07/2016	Tonsil (TON)	GD-3-TON	≈21-25 days	PP	+ (Seq-)	-	-	-	+	29.06	[5]
	Orosháza	11/2015	Tonsil (TON)	GD-5-TON	≈21-25 days	PP	-	-	+	30.77	+	22.75	[5]
	Orosháza	07/2016	Tonsil (TON)	GD-6-TON	≈35 days	AS	-	-	-	-	-	-	[5]
	Orosháza	03/2016	Lymph node-mesenteric (LN-MES)	GD-2-LN-MES	≈21-25 days	PP	+	31.11	-	33.15 (Seq+)	-	-	[5]
	Orosháza	07/2016	Lymph node-mesenteric (LN-MES)	GD-3-LN-MES	≈21-25 days	PP	-	31.63 (Seq+)	+	30.45	-	-	[5]
	Orosháza	07/2016	Lymph node-mesenteric (LN-MES)	GD-6-LN-MES	≈35 days	AS	-	-	+	25.27	-	-	[5]
	Orosháza	07/2016	Lymph node-submandibular (LN-SUB)	GD-4-LN-SUB	≈21-25 days	PP	-	35.37 (Seq-)	-	-	+ (Seq+)	-	[5]
	Orosháza	07/2016	Spleen (SPL)	GD-3-SPL	≈21-25 days	PP	+	30.97	-	-	-	-	[5]
	Orosháza	07/2016	Spleen (SPL)	GD-4-SPL	≈21-25 days	PP	+	26.96	-	-	-	-	[5]
	Orosháza	07/2016	Kidney (KID)	GD-3-KID	≈21-25 days	PP	+	28.56	-	-	-	-	[5]
	Orosháza	07/2016	Liver (LIV)	GD-3-LIV	≈21-25 days	PP	+	29.56	-	-	-	-	[5]
	Orosháza	05/2016	Liver (LIV)	GD-4-LIV	≈21-25 days	PP	+	31.65	-	-	-	-	[5]
	Orosháza	07/2016	Blood (SER)	GD-3-SER	≈21-25 days	PP	-	-	-	-	+	20.86	[5]

	Orosháza	07/2016	Urine (URI)	GD-3-URI	≈21-25 days	PP	+	31.10 (Seq+)	-	-	-	-	[5]

Table S2: Comparison of the clinical performances of triplex RT-qPCR (tRT-qPCR) and classical RT-PCR screening assays in N=142 archived RNA samples of swine (N=100 faecal/rectal swabs, N=1 urine and N=41 tissue specimens) which includes (but not limited to) known PSV, PTV and PoAstV-3 positives and negatives identified previously by our research group (see References of the samples column). All samples (including the previously tested specimens) were re-tested with PSV, PTV and PoAstV-3 RT-PCR screening assays prior to tRT-qPCR reactions. The table contains details of the analyzed animals/samples (AS: asymptomatic, PP: paraplegic) as well as results of RT-PCR (+: positive: visible band at the appropriate size range, -: negative: no visible band, or band(s) with non-appropriate size range) and RT-qPCR assays (-: negative: No Cq or Cq with >36.5; Samples with Cq values ≤ 36.5 were considered as RT-qPCR positive). Seq +: number of true-positive samples confirmed by Sanger-sequencing, Seq-: false positive (either non-viral sequences or non-sequenceable). Ambiguous samples (either RT-qPCR + / RT-PCR - or RT-qPCR - / RT-PCR +) are highlighted with red (PSV), green (PTV) or blue (PoAstV-3).

Farm ID	Sample ID	Location	Date (sampling)	Sample type	PSV-FAM	PTV-SUN	PoAstV3-Cy5
S105	S105_1	Sajószöged	5.24.2018	nasal swab	-	-	-
	S105_2	Sajószöged	5.24.2018	nasal swab	-	-	34.55
	S105_3	Sajószöged	5.24.2018	nasal swab	-	-	-
	S105_4	Sajószöged	5.24.2018	nasal swab	-	-	-
	S105_6	Sajószöged	5.24.2018	nasal swab	-	-	-
	S105_8	Sajószöged	5.24.2018	nasal swab	-	-	-
	S105_9	Sajószöged	5.24.2018	nasal swab	-	-	34.73
	S105_10	Sajószöged	5.24.2018	nasal swab	-	-	-
	S105_11	Sajószöged	5.24.2018	nasal swab	-	35.69	33.47
	S105_12	Sajószöged	5.24.2018	nasal swab	-	-	33.54
	S105_13	Sajószöged	5.24.2018	nasal swab	-	-	-
	S105_14	Sajószöged	5.24.2018	nasal swab	-	-	-
	S105_15	Sajószöged	5.24.2018	nasal swab	-	-	-
S126	S126_1	Szarvas	6.5.2018	nasal swab	-	-	-
	S126_2	Szarvas	6.5.2018	nasal swab	-	-	-
	S126_3	Szarvas	6.5.2018	nasal swab	-	-	-
	S126_5	Szarvas	6.5.2018	nasal swab	-	-	-
	S126_7	Szarvas	6.5.2018	nasal swab	-	-	-
	S126_8	Szarvas	6.5.2018	nasal swab	-	-	-
	S126_9	Szarvas	6.5.2018	nasal swab	-	-	-
	S126_10	Szarvas	6.5.2018	nasal swab	-	-	-
	S126_11	Szarvas	6.5.2018	nasal swab	-	-	-
	S126_12	Szarvas	6.5.2018	nasal swab	-	-	-
	S126_13	Szarvas	6.5.2018	nasal swab	-	-	-
	S126_14	Szarvas	6.5.2018	nasal swab	-	-	-
	S126_15	Szarvas	6.5.2018	nasal swab	-	-	-
S127	S127_1	Cibakháza	6.11.2018	nasal swab	-	-	34.76
	S127_2	Cibakháza	6.11.2018	nasal swab	-	-	-
	S127_3	Cibakháza	6.11.2018	nasal swab	-	-	-
	S127_5	Cibakháza	6.11.2018	nasal swab	-	-	-
	S127_10	Cibakháza	6.11.2018	nasal swab	-	-	-
	S127_11	Cibakháza	6.11.2018	nasal swab	-	-	33.76
	S127_12	Cibakháza	6.11.2018	nasal swab	-	-	34.00
	S127_13	Cibakháza	6.11.2018	nasal swab	-	32.72	-
	S127_14	Cibakháza	6.11.2018	nasal swab	-	-	-
	S127_15	Cibakháza	6.11.2018	nasal swab	-	-	-
S128	S128_1	Hajdúnánás	6.1.2018	nasal swab	-	-	-
	S128_2	Hajdúnánás	6.1.2018	nasal swab	-	-	-
	S128_4	Hajdúnánás	6.1.2018	nasal swab	-	-	34.13
	S128_5	Hajdúnánás	6.1.2018	nasal swab	-	-	36.11
	S128_6	Hajdúnánás	6.1.2018	nasal swab	-	-	-
	S128_7	Hajdúnánás	6.1.2018	nasal swab	-	-	34.66
	S128_10	Hajdúnánás	6.1.2018	nasal swab	-	-	-
	S128_11	Hajdúnánás	6.1.2018	nasal swab	-	-	-
	S128_12	Hajdúnánás	6.1.2018	nasal swab	-	-	-
	S128_13	Hajdúnánás	6.1.2018	nasal swab	-	-	34.61
	S128_14	Hajdúnánás	6.1.2018	nasal swab	-	-	-
	S128_15	Hajdúnánás	6.1.2018	nasal swab	-	-	-
S150	S150_1	Hajdószoboszló	6.14.2018	nasal swab	-	36.46	-
	S150_2	Hajdószoboszló	6.14.2018	nasal swab	-	-	-
	S150_3	Hajdószoboszló	6.14.2018	nasal swab	-	35.32	-
	S150_4	Hajdószoboszló	6.14.2018	nasal swab	-	35.00	-
	S150_5	Hajdószoboszló	6.14.2018	nasal swab	-	32.67	-
	S150_6	Hajdószoboszló	6.14.2018	nasal swab	-	36.36	-
	S150_7	Hajdószoboszló	6.14.2018	nasal swab	-	-	-
	S150_8	Hajdószoboszló	6.14.2018	nasal swab	-	-	-
	S150_9	Hajdószoboszló	6.14.2018	nasal swab	-	-	34.30
	S150_10	Hajdószoboszló	6.14.2018	nasal swab	-	36.47	35.23
	S150_11	Hajdószoboszló	6.14.2018	nasal swab	-	-	35.45
	S150_12	Hajdószoboszló	6.14.2018	nasal swab	-	-	-
	S150_13	Hajdószoboszló	6.14.2018	nasal swab	-	-	-
	S150_14	Hajdószoboszló	6.14.2018	nasal swab	-	35.10	-
	S150_15	Hajdószoboszló	6.14.2018	nasal swab	-	-	-
S151	S151_1	Kaba	6.12.2018	nasal swab	34.00	36.17	34.04
	S151_3	Kaba	6.12.2018	nasal swab	36.00	-	-
	S151_4	Kaba	6.12.2018	nasal swab	-	-	-
	S151_5	Kaba	6.12.2018	nasal swab	-	-	-
	S151_6	Kaba	6.12.2018	nasal swab	-	-	-
	S151_7	Kaba	6.12.2018	nasal swab	-	32.05	-
	S151_8	Kaba	6.12.2018	nasal swab	-	28.28	-
	S151_9	Kaba	6.12.2018	nasal swab	-	29.64	-

	S151_11	Kaba	6.12.2018	nasal swab	-	34.33	-
	S151_12	Kaba	6.12.2018	nasal swab	-	31.30	35.16
	S151_13	Kaba	6.12.2018	nasal swab	-	31.08	34.79
S152	S152_1	Karcag	6.13.2018	nasal swab	-	-	-
	S152_2	Karcag	6.13.2018	nasal swab	-	-	35.67
	S152_3	Karcag	6.13.2018	nasal swab	-	-	-
	S152_4	Karcag	6.13.2018	nasal swab	-	-	-
	S152_5	Karcag	6.13.2018	nasal swab	-	-	-
	S152_6	Karcag	6.13.2018	nasal swab	-	-	-
	S152_7	Karcag	6.13.2018	nasal swab	-	-	-
	S152_8	Karcag	6.13.2018	nasal swab	-	-	-
	S152_9	Karcag	6.13.2018	nasal swab	-	-	-
	S152_10	Karcag	6.13.2018	nasal swab	-	-	-
	S152_11	Karcag	6.13.2018	nasal swab	-	-	-
	S152_12	Karcag	6.13.2018	nasal swab	-	-	31.67
	S152_13	Karcag	6.13.2018	nasal swab	-	-	-
	S152_14	Karcag	6.13.2018	nasal swab	-	-	-
	S152_15	Karcag	6.13.2018	nasal swab	-	-	-
S153	S153_1	Biharnagybajom	6.15.2018	nasal swab	-	-	33.97
	S153_2	Biharnagybajom	6.15.2018	nasal swab	-	-	-
	S153_3	Biharnagybajom	6.15.2018	nasal swab	-	-	-
	S153_4	Biharnagybajom	6.15.2018	nasal swab	-	-	-
	S153_5	Biharnagybajom	6.15.2018	nasal swab	-	-	34.67
	S153_6	Biharnagybajom	6.15.2018	nasal swab	-	-	-
	S153_7	Biharnagybajom	6.15.2018	nasal swab	-	-	-
	S153_8	Biharnagybajom	6.15.2018	nasal swab	-	-	34.51
	S153_9	Biharnagybajom	6.15.2018	nasal swab	-	-	35.68
	S153_10	Biharnagybajom	6.15.2018	nasal swab	-	-	-
	S153_11	Biharnagybajom	6.15.2018	nasal swab	-	-	35.64
	S153_12	Biharnagybajom	6.15.2018	nasal swab	-	-	-
	S153_13	Biharnagybajom	6.15.2018	nasal swab	-	-	-
	S153_14	Biharnagybajom	6.15.2018	nasal swab	-	-	-
	S153_15	Biharnagybajom	6.15.2018	nasal swab	-	-	34.51
S154	S154_1	Nádudvar	6.11.2018	nasal swab	-	-	-
	S154_2	Nádudvar	6.11.2018	nasal swab	-	-	-
	S154_3	Nádudvar	6.11.2018	nasal swab	-	-	-
	S154_4	Nádudvar	6.11.2018	nasal swab	-	-	-
	S154_5	Nádudvar	6.11.2018	nasal swab	-	-	-
	S154_6	Nádudvar	6.11.2018	nasal swab	-	-	35.67
	S154_7	Nádudvar	6.11.2018	nasal swab	-	31.68	35.11
	S154_8	Nádudvar	6.11.2018	nasal swab	-	32.31	-
	S154_9	Nádudvar	6.11.2018	nasal swab	-	36.14	-
	S154_10	Nádudvar	6.11.2018	nasal swab	-	-	-
	S154_11	Nádudvar	6.11.2018	nasal swab	-	-	-
	S154_12	Nádudvar	6.11.2018	nasal swab	-	-	-
	S154_13	Nádudvar	6.11.2018	nasal swab	-	-	-
	S154_14	Nádudvar	6.11.2018	nasal swab	-	-	34.39
	S154_15	Nádudvar	6.11.2018	nasal swab	-	-	-
S155	S155_1	Nádudvar	6.15.2018	nasal swab	-	-	-
	S155_2	Nádudvar	6.15.2018	nasal swab	-	-	34.74
	S155_3	Nádudvar	6.15.2018	nasal swab	-	-	-
	S155_4	Nádudvar	6.15.2018	nasal swab	-	-	34.72
	S155_6	Nádudvar	6.15.2018	nasal swab	-	-	-
	S155_7	Nádudvar	6.15.2018	nasal swab	-	36.26	-
	S155_8	Nádudvar	6.15.2018	nasal swab	-	-	-
	S155_9	Nádudvar	6.15.2018	nasal swab	-	-	-
	S155_10	Nádudvar	6.15.2018	nasal swab	-	-	35.34
	S155_11	Nádudvar	6.15.2018	nasal swab	-	-	-
	S155_12	Nádudvar	6.15.2018	nasal swab	-	-	-
	S155_13	Nádudvar	6.15.2018	nasal swab	-	-	-
	S155_14	Nádudvar	6.15.2018	nasal swab	-	-	-
	S155_15	Nádudvar	6.15.2018	nasal swab	-	-	35.98
S156	S156_1	Püspökladány	6.12.2018	nasal swab	-	-	-
	S156_2	Püspökladány	6.12.2018	nasal swab	-	-	-
	S156_3	Püspökladány	6.12.2018	nasal swab	-	-	33.85
	S156_4	Püspökladány	6.12.2018	nasal swab	-	-	-
	S156_5	Püspökladány	6.12.2018	nasal swab	-	-	-
	S156_6	Püspökladány	6.12.2018	nasal swab	-	-	-
	S156_7	Püspökladány	6.12.2018	nasal swab	-	-	-
	S156_8	Püspökladány	6.12.2018	nasal swab	-	-	35.75
	S156_9	Püspökladány	6.12.2018	nasal swab	-	-	-
	S156_10	Püspökladány	6.12.2018	nasal swab	-	-	-

	S156_11	Püspökladány	6.12.2018	nasal swab	-	-	-
	S156_12	Püspökladány	6.12.2018	nasal swab	-	-	-
	S156_13	Püspökladány	6.12.2018	nasal swab	-	-	-
	S156_14	Püspökladány	6.12.2018	nasal swab	-	-	-
	S156_15	Püspökladány	6.12.2018	nasal swab	-	-	-
S157	S157_1	Füzesgyarmat	6.16.2018	nasal swab	34.17	33.60	-
	S157_2	Füzesgyarmat	6.16.2018	nasal swab	35.89	34.50	-
	S157_3	Füzesgyarmat	6.16.2018	nasal swab	-	-	-
	S157_4	Füzesgyarmat	6.16.2018	nasal swab	-	-	-
	S157_5	Füzesgyarmat	6.16.2018	nasal swab	-	33.54	-
	S157_6	Füzesgyarmat	6.16.2018	nasal swab	28.11	33.37	-
	S157_7	Füzesgyarmat	6.16.2018	nasal swab	33.92	-	-
	S157_8	Füzesgyarmat	6.16.2018	nasal swab	32.64	-	-
	S157_9	Füzesgyarmat	6.16.2018	nasal swab	-	36.39	34.60
	S157_10	Füzesgyarmat	6.16.2018	nasal swab	-	35.43	34.40
	S157_11	Füzesgyarmat	6.16.2018	nasal swab	-	-	-
	S157_12	Füzesgyarmat	6.16.2018	nasal swab	33.91	-	33.75
	S157_13	Füzesgyarmat	6.16.2018	nasal swab	29.10	-	-
	S157_14	Füzesgyarmat	6.16.2018	nasal swab	-	-	-
	S157_15	Füzesgyarmat	6.16.2018	nasal swab	34.43	-	-
S159	S159_1	Kocs	6.13.2018	nasal swab	-	-	34.80
	S159_2	Kocs	6.13.2018	nasal swab	-	-	-
	S159_3	Kocs	6.13.2018	nasal swab	-	-	-
	S159_4	Kocs	6.13.2018	nasal swab	-	-	-
	S159_5	Kocs	6.13.2018	nasal swab	-	-	-
	S159_7	Kocs	6.13.2018	nasal swab	-	-	-
	S159_8	Kocs	6.13.2018	nasal swab	-	-	-
	S159_9	Kocs	6.13.2018	nasal swab	-	-	35.82
	S159_10	Kocs	6.13.2018	nasal swab	-	-	-
	S159_11	Kocs	6.13.2018	nasal swab	-	-	32.61
	S159_12	Kocs	6.13.2018	nasal swab	-	-	-
	S159_13	Kocs	6.13.2018	nasal swab	-	-	-
	S159_14	Kocs	6.13.2018	nasal swab	-	-	-
	S159_15	Kocs	6.13.2018	nasal swab	-	-	-
S162	S162_2	Fadd	6.18.2018	nasal swab	32.92	36.26	33.87
	S162_4	Fadd	6.18.2018	nasal swab	-	-	-
	S162_5	Fadd	6.18.2018	nasal swab	32.73	36.50	-
	S162_6	Fadd	6.18.2018	nasal swab	33.41	-	-
	S162_7	Fadd	6.18.2018	nasal swab	-	-	35.07
	S162_11	Fadd	6.18.2018	nasal swab	-	-	-
	S162_12	Fadd	6.18.2018	nasal swab	36.24	-	34.24
	S162_13	Fadd	6.18.2018	nasal swab	-	-	-
	S162_14	Fadd	6.18.2018	nasal swab	-	-	-
S163	S163_1	Dunaszekcső	6.25.2018	nasal swab	32.68	-	-
	S163_2	Dunaszekcső	6.25.2018	nasal swab	35.50	-	-
	S163_3	Dunaszekcső	6.25.2018	nasal swab	35.17	-	-
	S163_4	Dunaszekcső	6.25.2018	nasal swab	-	-	-
	S163_5	Dunaszekcső	6.25.2018	nasal swab	-	-	-
	S163_6	Dunaszekcső	6.25.2018	nasal swab	-	-	-
	S163_7	Dunaszekcső	6.25.2018	nasal swab	-	-	-
	S163_8	Dunaszekcső	6.25.2018	nasal swab	-	-	-
	S163_9	Dunaszekcső	6.25.2018	nasal swab	-	-	-
	S163_10	Dunaszekcső	6.25.2018	nasal swab	-	-	-
S164	S164_1	Dávod	6.27.2018	nasal swab	30.90	34.94	-
	S164_2	Dávod	6.27.2018	nasal swab	35.80	-	34.05
	S164_3	Dávod	6.27.2018	nasal swab	-	-	-
	S164_4	Dávod	6.27.2018	nasal swab	-	-	-
	S164_5	Dávod	6.27.2018	nasal swab	-	-	34.55
	S164_6	Dávod	6.27.2018	nasal swab	-	-	-
	S164_7	Dávod	6.27.2018	nasal swab	-	-	-
	S164_8	Dávod	6.27.2018	nasal swab	-	-	33.73
	S164_9	Dávod	6.27.2018	nasal swab	-	35.52	33.89
	S164_10	Dávod	6.27.2018	nasal swab	-	-	-
	S164_11	Dávod	6.27.2018	nasal swab	-	-	-
	S164_12	Dávod	6.27.2018	nasal swab	-	-	-
	S164_13	Dávod	6.27.2018	nasal swab	-	-	-
	S164_14	Dávod	6.27.2018	nasal swab	-	-	-
	S164_15	Dávod	6.27.2018	nasal swab	-	-	34.04
S165	S165_1	Óalmás	6.28.2018	nasal swab	-	-	34.72
	S165_2	Óalmás	6.28.2018	nasal swab	-	-	-
	S165_3	Óalmás	6.28.2018	nasal swab	-	-	-
	S165_4	Óalmás	6.28.2018	nasal swab	-	-	-

	S165_5	Óalmás	6.28.2018	nasal swab	-	-	34.67
	S165_6	Óalmás	6.28.2018	nasal swab	-	28.27	-
	S165_7	Óalmás	6.28.2018	nasal swab	-	32.27	34.95
	S165_8	Óalmás	6.28.2018	nasal swab	-	34.52	-
	S165_9	Óalmás	6.28.2018	nasal swab	-	33.46	33.17
	S165_10	Óalmás	6.28.2018	nasal swab	-	33.88	-
	S165_11	Óalmás	6.28.2018	nasal swab	-	-	-
	S165_12	Óalmás	6.28.2018	nasal swab	-	34.08	-
	S165_13	Óalmás	6.28.2018	nasal swab	-	-	-
	S165_14	Óalmás	6.28.2018	nasal swab	-	-	-
	S165_15	Óalmás	6.28.2018	nasal swab	-	36.24	34.72
S166	S166_1	Somberek	6.27.2018	nasal swab	-	-	-
	S166_2	Somberek	6.27.2018	nasal swab	-	-	-
	S166_3	Somberek	6.27.2018	nasal swab	-	-	-
	S166_4	Somberek	6.27.2018	nasal swab	-	-	-
	S166_5	Somberek	6.27.2018	nasal swab	-	-	-
	S166_6	Somberek	6.27.2018	nasal swab	-	-	-
	S166_7	Somberek	6.27.2018	nasal swab	-	-	-
	S166_8	Somberek	6.27.2018	nasal swab	-	-	-
	S166_9	Somberek	6.27.2018	nasal swab	-	-	-
	S166_10	Somberek	6.27.2018	nasal swab	-	-	-
	S166_11	Somberek	6.27.2018	nasal swab	-	-	-
	S166_12	Somberek	6.27.2018	nasal swab	-	-	-
	S166_13	Somberek	6.27.2018	nasal swab	-	-	-
	S166_14	Somberek	6.27.2018	nasal swab	-	-	-
	S166_15	Somberek	6.27.2018	nasal swab	-	-	-
S167	S167_1	Mohács	6.25.2018	nasal swab	-	-	-
	S167_2	Mohács	6.25.2018	nasal swab	-	-	-
	S167_3	Mohács	6.25.2018	nasal swab	-	-	-
	S167_4	Mohács	6.25.2018	nasal swab	-	-	-
	S167_5	Mohács	6.25.2018	nasal swab	-	-	-
	S167_6	Mohács	6.25.2018	nasal swab	-	-	34.82
	S167_7	Mohács	6.25.2018	nasal swab	-	34.38	-
	S167_8	Mohács	6.25.2018	nasal swab	-	-	-
	S167_9	Mohács	6.25.2018	nasal swab	-	-	35.63
	S167_10	Mohács	6.25.2018	nasal swab	-	-	-
	S167_11	Mohács	6.25.2018	nasal swab	-	-	34.12
	S167_12	Mohács	6.25.2018	nasal swab	-	-	-
	S167_13	Mohács	6.25.2018	nasal swab	-	34.10	-
	S167_14	Mohács	6.25.2018	nasal swab	-	-	-
S168	S168_1	Újpetre	6.25.2018	nasal swab	-	-	-
	S168_2	Újpetre	6.25.2018	nasal swab	-	-	-
	S168_3	Újpetre	6.25.2018	nasal swab	-	-	-
	S168_4	Újpetre	6.25.2018	nasal swab	-	-	-
	S168_5	Újpetre	6.25.2018	nasal swab	-	-	-
	S168_6	Újpetre	6.25.2018	nasal swab	-	-	-
	S168_7	Újpetre	6.25.2018	nasal swab	-	-	-
	S168_8	Újpetre	6.25.2018	nasal swab	-	-	33.76
	S168_9	Újpetre	6.25.2018	nasal swab	-	-	-
	S168_10	Újpetre	6.25.2018	nasal swab	-	-	-
	S168_11	Újpetre	6.25.2018	nasal swab	-	-	-
	S168_12	Újpetre	6.25.2018	nasal swab	-	-	34.41
	S168_15	Újpetre	6.25.2018	nasal swab	-	-	-
S169	S169_1	Töttös	6.25.2018	nasal swab	-	-	-
	S169_2	Töttös	6.25.2018	nasal swab	-	33.69	-
	S169_3	Töttös	6.25.2018	nasal swab	-	36.17	34.71
	S169_4	Töttös	6.25.2018	nasal swab	-	-	-
	S169_5	Töttös	6.25.2018	nasal swab	-	-	-
	S169_6	Töttös	6.25.2018	nasal swab	-	-	-
	S169_7	Töttös	6.25.2018	nasal swab	-	-	-
	S169_8	Töttös	6.25.2018	nasal swab	-	-	-
	S169_9	Töttös	6.25.2018	nasal swab	-	-	34.65
	S169_10	Töttös	6.25.2018	nasal swab	-	-	-
	S169_11	Töttös	6.25.2018	nasal swab	-	-	-
	S169_12	Töttös	6.25.2018	nasal swab	-	-	-
	S169_13	Töttös	6.25.2018	nasal swab	-	-	-
	S169_14	Töttös	6.25.2018	nasal swab	-	-	-
	S169_15	Töttös	6.25.2018	nasal swab	-	-	-
S216	S216_1	Szarvas	6.28.2018	nasal swab	-	-	-
	S216_2	Szarvas	6.28.2018	nasal swab	-	-	-
	S216_3	Szarvas	6.28.2018	nasal swab	-	-	
	S216_5	Szarvas	6.28.2018	nasal swab	-	-	-

	S216_6	Szarvas	6.28.2018	nasal swab	-	-	-
	S216_7	Szarvas	6.28.2018	nasal swab	-	-	-
	S216_8	Szarvas	6.28.2018	nasal swab	-	-	-
	S216_9	Szarvas	6.28.2018	nasal swab	-	-	
	S216_10	Szarvas	6.28.2018	nasal swab	-	-	-
S234	S234_1	Dávod	9.20.2018	nasal swab	-	-	-
	S234_2	Dávod	9.20.2018	nasal swab	-	-	-
	S234_3	Dávod	9.20.2018	nasal swab	-	-	32.14
	S234_4	Dávod	9.20.2018	nasal swab	-	-	-
	S234_5	Dávod	9.20.2018	nasal swab	-	-	-
	S234_6	Dávod	9.20.2018	nasal swab	-	-	-
	S234_7	Dávod	9.20.2018	nasal swab	-	-	-
	S234_8	Dávod	9.20.2018	nasal swab	-	-	-
	S234_9	Dávod	9.20.2018	nasal swab	-	-	-
	S234_10	Dávod	9.20.2018	nasal swab	-	-	34.65
	S234_11	Dávod	9.20.2018	nasal swab	-	-	-
	S234_12	Dávod	9.20.2018	nasal swab	-	-	-
	S234_13	Dávod	9.20.2018	nasal swab	-	34.19	33.56
	S234_14	Dávod	9.20.2018	nasal swab	35.83	-	-
	S234_15	Dávod	9.20.2018	nasal swab	-	-	-
	S234_16	Dávod	9.20.2018	nasal swab	-	-	-
	S234_17	Dávod	9.20.2018	nasal swab	35.15	-	-
	S234_18	Dávod	9.20.2018	nasal swab	-	-	-
	S234_19	Dávod	9.20.2018	nasal swab	-	-	-
	S234_20	Dávod	9.20.2018	nasal swab	-	-	-
	S234_21	Dávod	9.20.2018	nasal swab	-	-	-
	S234_22	Dávod	9.20.2018	nasal swab	-	-	-
	S234_23	Dávod	9.20.2018	nasal swab	-	-	-
	S234_24	Dávod	9.20.2018	nasal swab	-	-	-
	S234_25	Dávod	9.20.2018	nasal swab	-	-	-
	S234_26	Dávod	9.20.2018	nasal swab	-	-	-
	S234_27	Dávod	9.20.2018	nasal swab	-	-	-
	S234_28	Dávod	9.20.2018	nasal swab	-	-	-
	S234_29	Dávod	9.20.2018	nasal swab	-	-	-
	S234_30	Dávod	9.20.2018	nasal swab	-	-	-
S240	S240_1	Töttös	9.26.2018	nasal swab	-	-	-
	S240_2	Töttös	9.26.2018	nasal swab	-	-	-
	S240_3	Töttös	9.26.2018	nasal swab	-	-	-
	S240_4	Töttös	9.26.2018	nasal swab	-	-	-
	S240_5	Töttös	9.26.2018	nasal swab	-	-	-
	S240_6	Töttös	9.26.2018	nasal swab	-	-	-
	S240_7	Töttös	9.26.2018	nasal swab	-	-	-
	S240_8	Töttös	9.26.2018	nasal swab	-	-	-
	S240_9	Töttös	9.26.2018	nasal swab	-	-	-
	S240_10	Töttös	9.26.2018	nasal swab	-	-	-
	S240_11	Töttös	9.26.2018	nasal swab	-	-	-
	S240_12	Töttös	9.26.2018	nasal swab	-	-	-
	S240_13	Töttös	9.26.2018	nasal swab	-	-	-
	S240_14	Töttös	9.26.2018	nasal swab	-	-	-
	S240_15	Töttös	9.26.2018	nasal swab	-	-	-
	S240_16	Töttös	9.26.2018	nasal swab	-	-	-
	S240_17	Töttös	9.26.2018	nasal swab	-	-	-
	S240_18	Töttös	9.26.2018	nasal swab	-	-	-
	S240_19	Töttös	9.26.2018	nasal swab	-	-	35.45
	S240_20	Töttös	9.26.2018	nasal swab	-	-	35.62
	S240_21	Töttös	9.26.2018	nasal swab	-	-	34.53
	S240_22	Töttös	9.26.2018	nasal swab	-	-	-
	S240_23	Töttös	9.26.2018	nasal swab	-	-	-
	S240_24	Töttös	9.26.2018	nasal swab	30.41	-	-
	S240_25	Töttös	9.26.2018	nasal swab	-	-	-
	S240_26	Töttös	9.26.2018	nasal swab	-	-	-
	S240_27	Töttös	9.26.2018	nasal swab	-	-	-
	S240_28	Töttös	9.26.2018	nasal swab	-	-	-
	S240_29	Töttös	9.26.2018	nasal swab	-	-	-
	S240_30	Töttös	9.26.2018	nasal swab	-	-	-
SZV	P1	Szigetvár	10.18.2017	nasal swab	-	-	-
	P2	Szigetvár	10.18.2017	nasal swab	-	-	-
	P3	Szigetvár	10.18.2017	nasal swab	34.77	-	-
	P4	Szigetvár	10.18.2017	nasal swab	-	-	-
	P5	Szigetvár	10.18.2017	nasal swab	-	-	-
	P6	Szigetvár	10.18.2017	nasal swab	-	-	-
	P7	Szigetvár	10.18.2017	nasal swab	-	-	-
	P8	Szigetvár	10.18.2017	nasal swab	-	-	-

	P9	Szigetvár	10.18.2017	nasal swab	-	-	-
	P10	Szigetvár	10.18.2017	nasal swab	35.49	-	-
	P11	Szigetvár	10.18.2017	nasal swab	-	-	-
	P12	Szigetvár	10.18.2017	nasal swab	-	-	-
	P13	Szigetvár	10.18.2017	nasal swab	33.78	-	-
	P14	Szigetvár	10.18.2017	nasal swab	34.77	-	-
	P15	Szigetvár	10.18.2017	nasal swab	31.07	-	-
	P16	Szigetvár	10.18.2017	nasal swab	-	-	-
	P17	Szigetvár	10.18.2017	nasal swab	-	-	-
	P18	Szigetvár	10.18.2017	nasal swab	-	-	-
	P19	Szigetvár	10.18.2017	nasal swab	-	-	-
	P20	Szigetvár	10.18.2017	nasal swab	-	-	-
	P21	Szigetvár	10.18.2017	nasal swab	-	-	-
	P22	Szigetvár	10.18.2017	nasal swab	-	-	-
	P23	Szigetvár	10.18.2017	nasal swab	-	-	-
	P24	Szigetvár	10.18.2017	nasal swab	-	-	-
	O3	Szigetvár	10.18.2017	nasal swab	33.13	-	-
	O4	Szigetvár	10.18.2017	nasal swab	-	-	-
	O7	Szigetvár	10.18.2017	nasal swab	-	-	34.80
	O11	Szigetvár	10.18.2017	nasal swab	-	-	34.44
	O21	Szigetvár	10.18.2017	nasal swab	-	-	-
BSZ	Ó-1_01	Bácsalmás	10.31.2017	nasal swab	-	-	-
	Ó-1_02	Bácsalmás	10.31.2017	nasal swab	-	-	-
	Ó-1_03	Bácsalmás	10.31.2017	nasal swab	-	-	-
	Ó-1_05	Bácsalmás	10.31.2017	nasal swab	-	-	-
	Ó-1_06	Bácsalmás	10.31.2017	nasal swab	-	-	34.02
	Ó-1_08	Bácsalmás	10.31.2017	nasal swab	-	-	35.08
	Ó-1_K	Bácsalmás	10.31.2017	nasal swab	-	-	-
	Ó-1083_01	Bácsalmás	10.31.2017	nasal swab	-	-	-
	Ó-1083_02	Bácsalmás	10.31.2017	nasal swab	-	-	-
	Ó-1083_03	Bácsalmás	10.31.2017	nasal swab	-	-	-
	Ó-1083_04	Bácsalmás	10.31.2017	nasal swab	-	-	-
	Ó-1083_05	Bácsalmás	10.31.2017	nasal swab	-	-	-
	Ó-1083_06	Bácsalmás	10.31.2017	nasal swab	-	-	-
	Ó-1083_07	Bácsalmás	10.31.2017	nasal swab	-	-	-
	Ó-1083_08	Bácsalmás	10.31.2017	nasal swab	-	-	35.08
	Ó-1083_09	Bácsalmás	10.31.2017	nasal swab	-	35.80	34.82
	Ó-1083_K	Bácsalmás	10.31.2017	nasal swab	-	-	-
	4752_02	Bácsalmás	10.31.2017	nasal swab	-	-	33.70
	4752_03	Bácsalmás	10.31.2017	nasal swab	-	-	-
	4752_04	Bácsalmás	10.31.2017	nasal swab	-	-	-
	4752_05	Bácsalmás	10.31.2017	nasal swab	-	-	-
	4752_06	Bácsalmás	10.31.2017	nasal swab	-	-	34.13
	4752_07	Bácsalmás	10.31.2017	nasal swab	-	-	-
	4752_08	Bácsalmás	10.31.2017	nasal swab	-	-	34.51
	4752_09	Bácsalmás	10.31.2017	nasal swab	-	-	-
	4752_K	Bácsalmás	10.31.2017	nasal swab	-	-	-
	4758_01	Bácsalmás	10.31.2017	nasal swab	-	-	-
	4758_02	Bácsalmás	10.31.2017	nasal swab	-	-	-
	4758_03	Bácsalmás	10.31.2017	nasal swab	-	-	-
	4758_05	Bácsalmás	10.31.2017	nasal swab	-	-	-
	4758_07	Bácsalmás	10.31.2017	nasal swab	-	-	-
	4758_08	Bácsalmás	10.31.2017	nasal swab	-	-	-
	4758_09	Bácsalmás	10.31.2017	nasal swab	-	-	-
	4758_K	Bácsalmás	10.31.2017	nasal swab	-	-	-
	4760_01	Bácsalmás	10.31.2017	nasal swab	-	-	-
	4760_02	Bácsalmás	10.31.2017	nasal swab	-	-	-
	4760_03	Bácsalmás	10.31.2017	nasal swab	-	-	-
	4760_04	Bácsalmás	10.31.2017	nasal swab	-	-	-
	4760_05	Bácsalmás	10.31.2017	nasal swab	-	-	-
	4760_07	Bácsalmás	10.31.2017	nasal swab	-	-	-
	4760_09	Bácsalmás	10.31.2017	nasal swab	-	35.89	-
	4760_K	Bácsalmás	10.31.2017	nasal swab	-	-	-
DSZ	DSZ-1_01K	Dunaszekcső	11.16.2017	nasal swab	-	-	-
	DSZ-1_02M	Dunaszekcső	11.16.2017	nasal swab	-	-	33.11
	DSZ-1_04M	Dunaszekcső	11.16.2017	nasal swab	-	-	-
	DSZ-1_05M	Dunaszekcső	11.16.2017	nasal swab	-	-	34.22
	DSZ-1_06M	Dunaszekcső	11.16.2017	nasal swab	-	-	-
	DSZ-1_08M	Dunaszekcső	11.16.2017	nasal swab	-	-	-
	DSZ-2_01	Dunaszekcső	11.16.2017	nasal swab	-	-	-
	DSZ-2_02M	Dunaszekcső	11.16.2017	nasal swab	-	-	-
	DSZ-2_03M	Dunaszekcső	11.16.2017	nasal swab	-	-	34.64
	DSZ-2_04M	Dunaszekcső	11.16.2017	nasal swab	-	-	-

	DSZ-2_05M	Dunaszekcső	11.16.2017	nasal swab	-	-	-
	DSZ-2_06M	Dunaszekcső	11.16.2017	nasal swab	-	-	-
	DSZ-2_07M	Dunaszekcső	11.16.2017	nasal swab	-	-	33.89
	DSZ-2_08M	Dunaszekcső	11.16.2017	nasal swab	-	-	-
	DSZ-3_01K	Dunaszekcső	11.16.2017	nasal swab	-	-	-
	DSZ-3_03M	Dunaszekcső	11.16.2017	nasal swab	-	-	34.04
	DSZ-3_04M	Dunaszekcső	11.16.2017	nasal swab	-	-	34.72
	DSZ-3_05M	Dunaszekcső	11.16.2017	nasal swab	-	-	-
	DSZ-3_06M	Dunaszekcső	11.16.2017	nasal swab	-	-	-
	DSZ-3_07M	Dunaszekcső	11.16.2017	nasal swab	-	-	-
	DSZ-4_01M	Dunaszekcső	11.16.2017	nasal swab	-	-	-
	DSZ-4_02M	Dunaszekcső	11.16.2017	nasal swab	-	35.84	-
	DSZ-4_03	Dunaszekcső	11.16.2017	nasal swab	-	-	-
	DSZ-4_04M	Dunaszekcső	11.16.2017	nasal swab	-	-	-
	DSZ-4_05	Dunaszekcső	11.16.2017	nasal swab	-	-	-
	DSZ-4_06M	Dunaszekcső	11.16.2017	nasal swab	-	-	-
	DSZ-5_01M	Dunaszekcső	11.16.2017	nasal swab	-	-	-
	DSZ-5_02M	Dunaszekcső	11.16.2017	nasal swab	-	-	-
	DSZ-5_03M	Dunaszekcső	11.16.2017	nasal swab	-	-	-
	DSZ-5_04M	Dunaszekcső	11.16.2017	nasal swab	-	-	-
	DSZ-5_06M	Dunaszekcső	11.16.2017	nasal swab	-	-	-
GD	GD_01	Orosháza	9.15.2016	nasal swab	-	-	-
	GD_02	Orosháza	9.15.2016	nasal swab	34.52	-	-
	GD_03	Orosháza	9.15.2016	nasal swab	31.43	-	-
	GD_04	Orosháza	9.15.2016	nasal swab	36.41	-	-
	GD_05	Orosháza	9.15.2016	nasal swab	31.68	-	35.37
	GD_06	Orosháza	9.15.2016	nasal swab	31.13	-	34.42
	GD_07	Orosháza	9.15.2016	nasal swab	31.15	-	35.07
	GD_08	Orosháza	9.15.2016	nasal swab	-	-	-
	GD_09	Orosháza	9.15.2016	nasal swab	33.19	35.56	-
	GD_10	Orosháza	9.15.2016	nasal swab	34.45	-	-
	GD0717_1A	Orosháza	6.07.2017	nasal swab	34.65	35.65	-
	GD0717_2A	Orosháza	6.07.2017	nasal swab	-	-	-
	GD0717_3A	Orosháza	6.07.2017	nasal swab	-	-	-
	GD0717_1	Orosháza	6.07.2017	nasal swab	-	-	-
	GD0717_2	Orosháza	6.07.2017	nasal swab	-	-	-
	GD0717_4	Orosháza	6.07.2017	nasal swab	-	-	-
	GD0717_5	Orosháza	6.07.2017	nasal swab	-	-	-
	GD0717_7	Orosháza	6.07.2017	nasal swab	-	-	-
	GD0717_8	Orosháza	6.07.2017	nasal swab	-	-	-

Table S3: Results of porcine sapelovirus (PSV), poricne teschovirus (PTV) and porcine astrovirus type 3 (PoAstV3) RT-qPCR assays of N=473 nasal swabs collected in N=28 different Hungarian swine farms. FAM/SUN/Cy5: detection channels of the given virus. The table contains details of the farms/samples as well as measured Cq values (≤ 36.5). (-: negative: No Cq or Cq with >36.5; Samples with Cq values ≤ 36.5 were considered as RT-qPCR positive).

Farm ID	Location	Date (sampling)	Sample ID	Sample type	Health status	PSV-FAM	PTV-SUN	PoAstV3-Cy5
MB	Bodony	20.03.2020	MB-H-Agy	Brain stem	paraplegic	-	-	-
SS	Somogysárd	30.09.2019	SS21-GV-Ny	Spinal cord	paraplegic	-	-	-
	Somogysárd	30.09.2019	SS22-GV-Ny	Spinal cord	paraplegic	-	-	-
	Somogysárd	30.09.2019	SS21-GV-H	Spinal cord	paraplegic	-	-	-
	Somogysárd	30.09.2019	SS22-GV-H	Spinal cord	paraplegic	-	-	-
	Somogysárd	30.09.2019	SS21-GV-K	Spinal cord	paraplegic	-	-	-
	Somogysárd	30.09.2019	SS22-GV-K	Spinal cord	paraplegic	-	-	-
	Somogysárd	30.09.2019	SS21-KV	Cerebellum	paraplegic	-	-	-
	Somogysárd	30.09.2019	SS22-KV	Cerebellum	paraplegic	-	-	-
	Somogysárd	30.09.2019	SS21-AV	Brain stem	paraplegic	-	-	-
	Somogysárd	30.09.2019	SS22-AV	Brain stem	paraplegic	-	-	-
OR	Orosháza	25.05.2021	OR-B1-KA	Cerebellum	paraplegic	-	-	-
	Orosháza	25.05.2021	OR-B2-KA	Cerebellum	paraplegic	-	-	-
	Orosháza	25.05.2021	OR-B3-KA	Cerebellum	paraplegic	-	-	-
	Orosháza	25.05.2021	OR-B4-KA	Cerebellum	paraplegic	-	-	-
	Orosháza	25.05.2021	OR-B5-KA	Cerebellum	paraplegic	-	-	-
	Orosháza	25.05.2021	OR-B1-AGY	Brain stem	paraplegic	-	-	-
	Orosháza	25.05.2021	OR-B2-AGY	Brain stem	paraplegic	-	-	-
	Orosháza	25.05.2021	OR-B3-AGY	Brain stem	paraplegic	-	-	-
	Orosháza	25.05.2021	OR-B4-AGY	Brain stem	paraplegic	-	-	-
	Orosháza	25.05.2021	OR-B5-AGY	Brain stem	paraplegic	-	-	-

Table S4: Results of porcine sapelovirus (PSV), porcine teschovirus (PTV) and porcine astrovirus type 3 (PoAstV3) RT-qPCR assays of N=21 samples from various sites of the central nervous system of N= 8 paraplegic pig with unknown aetiology. FAM/SUN/Cy5: detection channels of the given virus. The table contains details of the farms/samples as well as measured Cq values (≤ 36.5). (-: negative: No Cq or Cq with >36.5; Samples with Cq values ≤ 36.5 were considered as RT-qPCR positive).
