# Supplementary material for: Development and Large-Scale Testing of a Novel One-Step Triplex RT-qPCR Assay for Simultaneous Detection of “Neurotropic” Porcine Sapeloviruses, Teschoviruses (Picornaviridae) and Type 3 Porcine Astroviruses (Astroviridae) in Various Samples including Nasal Swabs
Source: Viruses. 2022 Mar 2;14(3):513. doi: 10.3390/v14030513 (PMC8952109; doi:10.3390/v14030513)
Supplement: Supplementary file 1 [file viruses-14-00513-s001.zip › Technical appendix - viruses-1487148.pdf]

Technical appendix

Detailed description of the production process of PSV, PTV and PoAstV-3 RNA standards

The different RNA standards were *in vitro* prepared to optimize the RT-qPCR reaction conditions and the assay performance. First, two archived samples were selected which are containing the viruses of interest (PSV&PTV positive B1M faecal sample and PoAstV-3 positive GD3-brainstem sample) with known genome sequences (PSV: MN807752, PTV: MN807750; PoAstV-3: KY073231) determined previously [5,10]. Based on the known virus sequences, sequence-specific oligonucleotide primer pairs were designed to produce short (406 – 591 bp) PCR-products that contain the target sites of primers/probes used for virus-specific RT-qPCR assays (Table 1). The RT-products were generated using M-MLV-RT reverse transcriptase and DreamTaq polymerase (Thermo-Fisher, Waltham, MA, USA) enzymes and the same reaction conditions and reagents described previously [30]. The generated PCR products were separated by agarose gel-electrophoresis and purified using GeneJET gel extraction kit (Thermo-Fisher) according to the manufacturer's instructions. The concentration/purity of the purified PCR-products was measured using NanoDrop 2000 spectrophotometer (Thermo-Fisher). Approximately 5ng of the PCR products were ligated into T7-promoter sequence-containing pCR2.1 vectors of TA Cloning® Kit (Thermo-Fisher) following the manufacturer's instructions. DH5-Alpha-type competent Escherichia coli cells (Thermo-Fisher) were transformed by ligated pCR2.1 plasmids and cultured overnight in 100µg/ml ampicillin-containing Luria-Bertani (LB) agar plates. The presence and orientation of inserts in selected clones were checked by colony PCR using insert-specific reverse primers and M13-universal forward primer. Clones with proper orientation of the inserts were selected and cultured overnight in 100µg/ml ampicillin-containing LB media. Plasmids were isolated from the overnight cultures by Zyppy Plasmid Miniprep Kit (Zymo Research, USA) according to the manufacturer's instructions and their concentration/purity was measured using NanoDrop 2000 (Thermo-Fisher). The inserts of the selected purified plasmids were sequenced directly by Sanger sequencing. Selected plasmids with non-mutated virus-specific inserts were linearized using HindIII (PSV-, and PTV-insert containing plasmids) or BamHI (PoAstV-3-insert containing plasmids) restriction enzymes (Thermo-Fisher) according to the manufacturer's instructions. C.a. 200ng of linearized plasmid was used as templates for in vitro RNA synthesis using TranscriptAid T7 High Yield Transcription Kit (Thermo-Fisher) according to the protocol provided by the manufacturer. The resulting T7 reaction products were digested and purified with a total of three consecutive series of TURBO™ DNase enzyme digestion (Thermo-Fisher) and RNA extraction by TRI® Reagent (MRC, USA) following the instruction provided by the manufacturer. The presence of plasmid DNA and in vitro transcribed viral RNA was tested by (RT-)PCR method with or without the use of RT enzyme at the end of each digestion-purification series. The concentration/purity of the purified, plasmid DNA-free RNA samples was measured using NanoDrop 2000 and Qubit 4 Fluorometer (Thermo-Fisher). The copy number of the produced viral RNA standards were calculated from the measured concentrations and the known molecular weights (calculated from the nt sequence) of the products. Ten-fold serial dilutions of mixed viral RNA standards (from 2×10^9 to 2.0 copies/µl of each element of the mixture) were created using NFW.
